# The Entomopathogenic Fungus Beauveria bassiana Employs Autophagy as a Persistence and Recovery Mechanism during Conidial Dormancy

**DOI:** 10.1128/mbio.03049-22

**Published:** 2023-02-21

**Authors:** Jin-Li Ding, Hai-Yan Lin, Jia Hou, Ming-Guang Feng, Sheng-Hua Ying

**Affiliations:** a Institute of Microbiology, College of Life Sciences, Zhejiang University, Hangzhou, China; The Sainsbury Laboratory, Norwich Research Park; Max Planck Institute for Terrestrial Microbiology

**Keywords:** Cvt pathway, filamentous fungus, aspartyl aminopeptidase, autophagy, conidial aging, environmental persistence

## Abstract

Many filamentous fungi develop a conidiation process as an essential mechanism for their dispersal and survival in natural ecosystems. However, the mechanisms underlying conidial persistence in environments are still not fully understood. Here, we report that autophagy is crucial for conidial lifespans (i.e., viability) and vitality (e.g., stress responses and virulence) in the filamentous mycopathogen Beauveria bassiana. Specifically, Atg11-mediated selective autophagy played an important, but not dominant, role in the total autophagic flux. Furthermore, the aspartyl aminopeptidase Ape4 was found to be involved in conidial vitality during dormancy. Notably, the vacuolar translocation of Ape4 was dependent on its physical interaction with autophagy-related protein 8 (Atg8) and associated with the autophagic role of Atg8, as determined through a truncation assay of a critical carboxyl-tripeptide. These observations revealed that autophagy acted as a subcellular mechanism for conidial recovery during dormancy in environments. In addition, a novel Atg8-dependent targeting route for vacuolar hydrolase was identified, which is essential for conidial exit from a long-term dormancy. These new insights improved our understanding of the roles of autophagy in the physiological ecology of filamentous fungi as well as the molecular mechanisms involved in selective autophagy.

## INTRODUCTION

The environmental persistence of pathogens is a key attribute related to their potential to initiate epidemics in sensitive host populations; that is, the longer a pathogen survives, the more likely it can infect a host (1, 2). Filamentous pathogenic fungi generate abundant asexual spores as infective agents. After maturation, spores are disconnected from mother cells and enter a dormant state to promote fungal dissemination and environmental survival (3, 4). Neurospora crassa is a model filamentous fungus that produces conidia through asexual sporulation. N. crassa conidia undergo oxidation-dependent aging during their persistence in environments. Accordingly, antioxidant enzymes (e.g., catalase and superoxide dismutase) and metabolites (e.g., ergothioneine) significantly contribute to conidial longevity (5, 6). Nevertheless, the current understanding of conidial persistence postmaturation remains incomplete, particularly at the subcellular level. Macroautophagy (hereafter autophagy) is a conserved mechanism for maintaining intracellular homeostasis by removing excess or damaged cytoplasmic components (i.e., proteins and organelles) in lysosomes/vacuoles, including through both nonselective and selective mechanisms (7). Autophagy in filamentous fungi has been linked to numerous physiological processes, including development, virulence, and hyphal aging (8–10). However, the role of autophagy in the environmental persistence of spores remains unclear.

Filamentous insect-pathogenic fungi such as Beauveria bassiana and Metarhizium anisopliae represent emerging research systems for investigating host-fungus interactions (11) and have also been extensively used in the biological control of insect pests (12). Their conidia act as infective propagules within environments due to their dispersal capacity (13). In addition, conidia can practically function as active agents for the biological control of insect pests (12). Thus, longer conidial persistence leads to higher biocontrol efficacy. However, detailed understandings of mechanisms ensuring conidial longevity for insect-pathogenic fungi remain scarce. Given the importance of understanding environmental persistence in basic and applied research, B. bassiana could represent an ideal pathogenic filamentous fungus for exploring mechanisms involved in postmaturation conidial persistence.

Autophagy and its associated genes are involved in diverse physiological traits in B. bassiana (14, 15). Over 40 autophagy-related genes (ATGs) have been implicated in the autophagy pathway, 18 of which are considered core genes indispensable for autophagic processes, while others are required for specific autophagic pathways (16). All core ATGs are conserved in entomopathogenic fungi (17). Autophagy-related protein 1 (Atg1; a serine/threonine protein kinase) and Atg8 (a ubiquitin-like protein) are necessary for autophagosome formation in B. bassiana, and Atg11 acts as a scaffold protein in selective autophagy that contributes to the fungal stress response (e.g., oxidative and oligotrophic stresses), conidiation, and virulence (14, 15). We consequently hypothesized that autophagy might be a safeguard mechanism for postmaturation conidial survival in B. bassiana.

In the present study, autophagy was shown to be required for conidial viability and vitality (e.g., stress response and virulence) during long-term survival. Further, aspartyl aminopeptidase (Ape4) was observed to contribute to conidial survival and was translocated from the cytoplasm into vacuoles via direct interaction with Atg8. To our knowledge, this is the first report to reveal the importance of autophagy in conidial lifespans while also documenting a novel intracellular trafficking route for vacuolar hydrolases during conidial recovery from dormancy.

## RESULTS

### Autophagy functions as a persistence mechanism during conidial dormancy.

**FIG 1:**
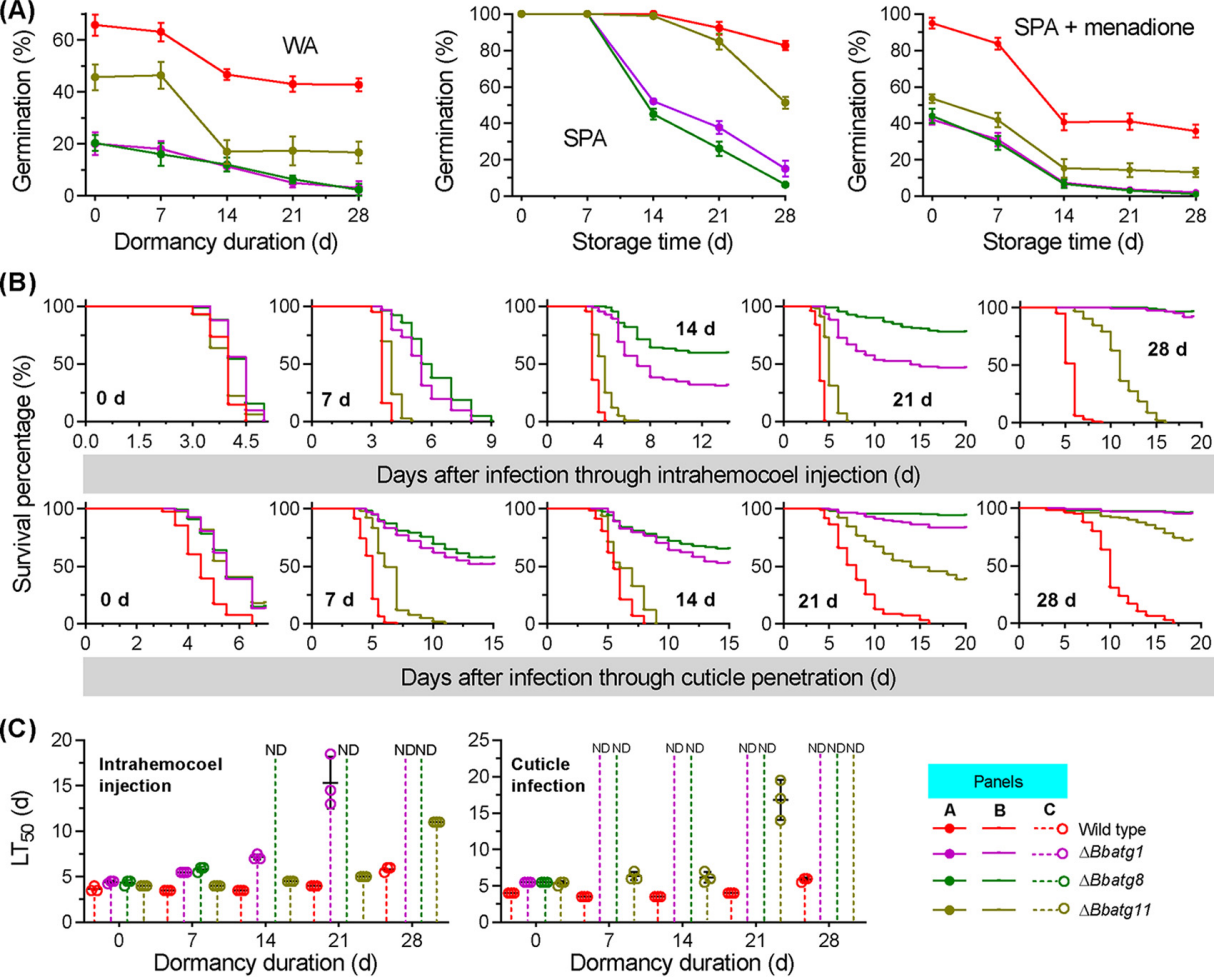
Phenotypic assays for Δ*Bbatg1*, Δ*Bbatg8*, and Δ*Bbatg11* mutant strains. Conidia were stored at 25°C and sampled at 7-day intervals for up to 4 weeks. (A) Conidial germination. Sampled conidia were inoculated on water agarose (WA), nutrient-rich (SPA), and menadione-stress (SPA plus menadione) plates. After 24 h of incubation at 25°C, conidial germination was determined and indicated as the germination percentage (%). Intrahemocoel injection and cuticle penetration bioassay tests were used to determine conidial virulence. (B) Survival trends (%) of the infected insect hosts across bioassays. Statistical differences between paired curves were calculated using the log-rank test and were considered significant at the *P < *0.05 level. (C) Median lethal time (LT_50_) was calculated using the Kaplan-Meier method for three independent bioassays of conidia at the indicated age point. ND, LT_50_ values were not determined due to mortalities of <50%; d, days.

Conidia (7 days old) from all gene disruption mutants exhibited significantly reduced germination rates on water agarose (WA) plates (Fig. 1A) (*F_3_*_,_*_8_* = 138.8, *P < *0.01) compared to the germination percentage (GP) for the WT strain of 65.7 ± 4.0% (mean ± standard deviation [SD]). The Δ*Bbatg11* mutant exhibited a GP of 45.7 ± 4.9%, which was significantly higher than that of the Δ*Bbatg1* and Δ*Bbatg8* mutant strains (18.0 ± 1.0% and 22.0% ± 1.0%, respectively). After 28 days of dormancy (DOD), almost all conidia of the Δ*Bbatg1* and Δ*Bbatg8* mutants lost viability, while the WT and Δ*Bbatg11* mutant strain conidia exhibited GPs of 43.3 ± 1.5% and 19.0 ± 1.0%, respectively. Significant differences in GP were not detected between the WT and the Δ*Bbatg1* or Δ*Bbatg8* mutants that were dormant for 7 days on nutrient-rich sucrose-peptone agar (SPA) plates. The GP of the Δ*Bbatg11* mutant conidia was not significantly different from that of the WT strain conidia that were dormant up to 14 days. A significant difference was observed for the GPs between the WT and Δ*Bbatg11* mutant strains after these time points. Thiazolyl blue tetrazolium bromide (MTT) assays also indicated that the conidial viability for autophagy-null mutants declined during dormancy, consistent with the variation in GP values (see Fig. S1A in the supplemental material). The germination of the Δ*Bbatg11* mutant conidia was significantly delayed after 14 and 21 DOD, but their GPs did not significantly differ from that of the WT strain (Fig. S1B). All mutant conidia exhibited significantly reduced GPs for 7-day-old conidia under oxidative stress (Fig. 1A; *F_3_*_,_*_8_* = 363.4, *P < *0.01) compared to WT strain conidia (96.7 ± 1.2%). The GP of the Δ*Bbatg11* mutant conidia was 53.7 ± 2.5%, representing a higher value than those of the Δ*Bbatg1* and Δ*Bbatg8* mutant strains (43.0 ± 2.0% and 44.7 ± 3.0%, respectively). At the end of incubation, the GP values of Δ*Bbatg1* and Δ*Bbatg8* conidia decreased by 95.3% and 97.7%, respectively, compared to their newly generated conidia. The GP reductions for the WT and the Δ*Bbatg11* mutant strain conidia were 53.7% and 73.9%, respectively. The morphologies of the germlings grown under the above-described three conditions are shown in Fig. S2A.

10.1128/mbio.03049-22.3 FIG S1 Analyses of conidial viability. (A) Fungal strains were cultured on SDAY plates for 7 days until conidiation, and conidia were stored at 0, 7, 14, 21, and 28 days. The sampled conidia were stained with 3-(4,5-dimethylthiazol-2-yl)-2,5-diphenyltetrazolium bromide (MTT). The resultant MTT-formazan was dissolved in dimethyl sulfoxide, and the absorbance of solution was examined at 579 nm. Fungal strains were cultured on SDAY plates for 7 days until conidiation, and conidia were stored at 0, 7, 14, 21, and 28 days. The sampled conidia were inoculated on nutrient-rich plates (SPA) and incubated at 25°C. Conidial germination was determined every two hours for 24 h and indicated as the germination percentage (%). (B) The wild-type and autophagy-null strains; (C) the wild-type, Δ*Bbape4*, and its complementation mutant strains; (D) the wild-type, Δ*Bbatg8*, and Δ*Bbatg8^A8T^* strains. Download FIG S1, PDF file, 0.4 MB. Copyright © 2023 Ding et al. 2023 Ding et al. https://creativecommons.org/licenses/by/4.0/ This content is distributed under the terms of the Creative Commons Attribution 4.0 International license.

10.1128/mbio.03049-22.4 FIG S2 Microscopic view of conidial germination. Fungal strains were cultured on SDAY plates for 7 days until conidiation, and conidia were stored at 0, 7, 14, 21, and 28 days. The sampled conidia were inoculated on water agar plates (WA), nutrient-rich plates (SPA), and stress plates (SPA plus menadione). After an incubation of 24 h at 25°C, morphologies of fungal cells were recorded. Bars = 10 μm. (A) The wild-type, Δ*Bbatg1*, Δ*Bbatg8*, and Δ*Bbatg11* mutant strains; (B) the wild-type and complementation mutants for the Δ*Bbatg1*, Δ*Bbatg8*, and Δ*Bbatg11* mutant strains; (C) the wild-type, Δ*Bbape4*, and its complementation mutant strains; (D) the wild-type, Δ*Bbatg8*, and Δ*Bbatg8^A8T^* strains; (E) *BbATG8* and truncated *BbATG8* (*BbATG8^T^*) was transformed into the wild-type strain (WT), and the resultant strains were named WT^A8^ and WT^A8T^, respectively. Download FIG S2, PDF file, 3.0 MB. Copyright © 2023 Ding et al. 2023 Ding et al. https://creativecommons.org/licenses/by/4.0/ This content is distributed under the terms of the Creative Commons Attribution 4.0 International license.

Two types of inoculation methods were used to assay the infectiousness of conidia (Fig. 1B) and were then compared (Table S1). In the intrahemocoel injection assay, 7-day-old conidia from the four strains killed all insects. The host survival curve for the Δ*Bbatg8* strain conidia was significantly different from those of the WT and Δ*Bbatg11* mutant strains (*P < *0.0001) but did not differ from that of the Δ*Bbatg1* mutant (*P = *0.5492). As incubation time increased, the survival trends of hosts for the three disruptants significantly differentiated from each other and from that of the WT strain conidia. After 28 DOD, the insects infected with the Δ*Bbatg1* or *Bbatg8* mutant conidia exhibited very low mortality (<5%). Both the WT and Δ*Bbatg11* mutant strains killed all insects, but their associated survival curves significantly differed (*P < *0.0001).

10.1128/mbio.03049-22.1 TABLE S1 *P* values from log-rank test for the paired survival curves in the bioassay. Download Table S1, DOCX file, 0.02 MB. Copyright © 2023 Ding et al. 2023 Ding et al. https://creativecommons.org/licenses/by/4.0/ This content is distributed under the terms of the Creative Commons Attribution 4.0 International license.

In the cuticle infection assay, no significant differences were observed among the host survival curves for the three gene disruption mutants after applying the 7-day-old conidia (i.e., newly formed conidia). The host survival trends corresponding to the three disruptants significantly differentiated with increased incubation time, and their survival curves significantly differed from that of the WT strain at any sampling time point. At 21 DOD, both the Δ*Bbatg1* and Δ*Bbatg8* mutant strains caused very low mortality (<5%), and the mortality caused by Δ*Bbatg11* was <30%. The median lethal time (LT_50_) values for the three disruptants increased with increasing incubation time (Fig. 1C). For the intrahemocoel injection assay, the LT_50_ at 5.8 days was calculated for WT conidia after 28 DOD. LT_50_ values were not calculated for the conidia of Δ*Bbatg1* and Δ*Bbatg8* mutants, because their cumulative host mortalities were <50%, while the LT_50_ value for Δ*Bbatg11* conidia was 11 days. Further, LT_50_ values were not calculated for all disruptants for the cuticle infection assays of conidia that were dormant for 28 days. The significant differences were not observed in the indicated phenotypes between wild-type and complementation mutant strains (Fig. S2B and Fig. S3A to C).

10.1128/mbio.03049-22.5 FIG S3 Phenotypic evaluation. Conidia of the indicated strain were stored at 25°C and sampled at an interval of 7 days to 4 weeks. (A, D, and G) Conidial germination. The sampled conidia were inoculated on water agarose (WA), nutrient-rich (SPA), and menadione-stress (SPA plus menadione) plates. After 24 h of incubation at 25°C, conidial germination was determined and indicated as the germination percentage (%). Two types of bioassay methods (i.e., intrahemocoel injection and cuticle penetration) were used to determine conidial virulence. (B, E, and H) Survival trends (%) of the infected insect host over bioassay time. The statistical difference between the paired curves was calculated by the log-rank test and was considered to be significant at *P < *0.05. (C, F, and I) The median lethal time (LT_50_) was calculated using the Kaplan-Meier method for three independent bioassays of conidia at the indicated age point. ND, no LT_50_ value was determined due to mortalities of less than 50%. (A, B, and C) Assays for the complementation mutant strains. The Δ*Bbatg1*, Δ*Bbatg8*, and Δ*Bbatg11* mutant strains were complemented by ectopic insertion of their entire genes. (D, E, and F) Functionality of mutated BbApe4 was determined in the Δ*Bbape4* mutant strain, using the wild-type strain as the positive control. T3 to T7, BbApe4 with a single mutation at different motifs (T3 to T7). (G, H, and I) Phenotypic assays for the strain overexpressing the gene *BbATG8* or *BbATG8^T^*. *BbATG8* and truncated *BbATG8* (*BbATG8^T^*) were transformed into the wild-type strain (WT), and the resultant strains were named WT^A8^ and WT^A8T^, respectively. Download FIG S3, PDF file, 0.8 MB. Copyright © 2023 Ding et al. 2023 Ding et al. https://creativecommons.org/licenses/by/4.0/ This content is distributed under the terms of the Creative Commons Attribution 4.0 International license.

### Autophagy occurs during conidial germination.

**FIG 2:**
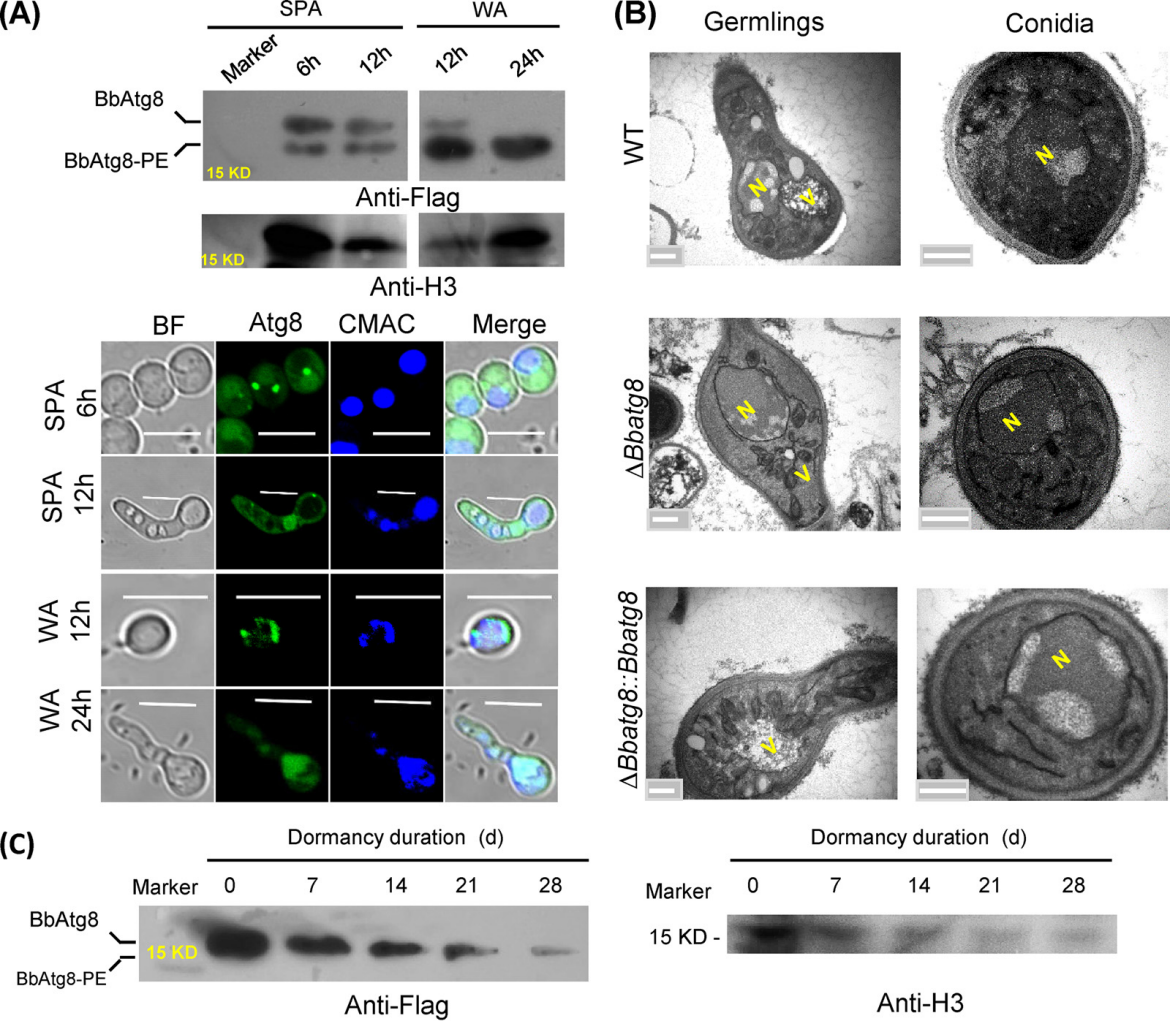
Autophagy during conidial germination. To assess autophagy during conidial germination, the wild-type (WT) strain was transformed with the fusion gene *3× Flag-BbATG8*. Conidia from the resultant strain were inoculated on nutrient-rich (SPA) and water agarose (WA) plates, followed by incubation at 25°C. Germlings were sampled at the indicated time points. (A) Lipidation of BbAtg8 was detected with immunoblotting analyses using the anti-Flag antibody and with histone (H3) used as the control. PE, phosphatidylethanolamine. Autophagic flux was determined by fusing GFP to the amino terminus of BbAtg8. Vacuoles were identified by staining fungal cells with the fluorescent dye CMAC. Bars = 5 μm. (B) Transmission electron microscopy was used to examine ultrastructures in germlings and conidia. N, nucleus, V, vacuole. Bars = 0.2 μm. (C) Protein state of BbAtg8 in dormant conidia. Conidia of the above-described transformant were stored at 25°C for 4 weeks. Aliquots (100 mg) of conidia were sampled at the indicated time point, and the protein state of BbAtg8 was examined with immunoblotting analyses, as described above. d, days. Histone 3 (H3) was used as the control d, days.

BbAtg8 lipidation was observed during conidial germination on SPA and WA plates (Fig. 2A). Furthermore, green fluorescent protein (GFP)-tagged BbAtg8 exhibited aggregated signals and then gradually translocated into vacuoles. Transmission electron microscopy (TEM) indicated that vacuoles were not observed in conidia but were observed in germlings. Δ*Bbatg8* germlings exhibited significantly reduced vacuoles (Fig. 2B). Immunoblotting analyses indicated that BbAtg8 lipidation was not observed in conidia during dormancy (Fig. 2C). These results suggest that autophagy does not occur during conidial dormancy, but is observed in germinating conidia.

*BbATG1*, *BbATG8*, and *BbATG11* exhibited transcriptional variability during conidial germination, vegetative growth, and pathogenic growth (Fig. S4A). The autophagy inhibitor (3-methyladenine [3-MA]) and activator (rapamycin [RA]) both inhibited conidial germination (Fig. S4B and C). In addition, the two chemicals repressed and enhanced autophagy, as indicated by autophagosome formation and BbAtg8 lipidation, respectively (Fig. S4D). Thus, these results suggest that autophagy is finely tuned during conidial germination.

10.1128/mbio.03049-22.6 FIG S4 Autophagy homeostasis is critical for conidial germination. (A) Transcriptional analyses of autophagy-related genes (*ATG*) 1, 8, and 11 were performed in B. bassiana during conidial germination, vegetative growth, and pathogenic growth. (B) Conidial germination was examined on water agar plates (WA), nutrient-rich plates (SPA), and stress plates (SPA plus menadione [M]). Effects of autophagy inhibitor and activator on conidial germination were determined by adding 3-methyladenine (3-MA) and rapamycin (RA), respectively, into the indicated medium. (C) The germination percentage was examined at 24 h postincubation, and morphologies of germinating cells were recorded. Bars = 10 μm. (D) The transgenic strain expressing *GFP-BbATG8* was cultured on SDAY plates with 3-MA or RA. The fluorescent dyes CMAC and GFP-BbAtg8 were used to indicate vacuole and autophagic flux, respectively. Lipidation of BbAtg8 was detected with immunoblotting analyses, using histone 3 (H3) as the control. The wild type was used to determine the specificity of anti-Flag antibody. Bars = 5 μm. Download FIG S4, PDF file, 2.5 MB. Copyright © 2023 Ding et al. 2023 Ding et al. https://creativecommons.org/licenses/by/4.0/ This content is distributed under the terms of the Creative Commons Attribution 4.0 International license.

### BbAtg8 translocates BbApe4 into vacuoles via direct interaction.

**FIG 3:**
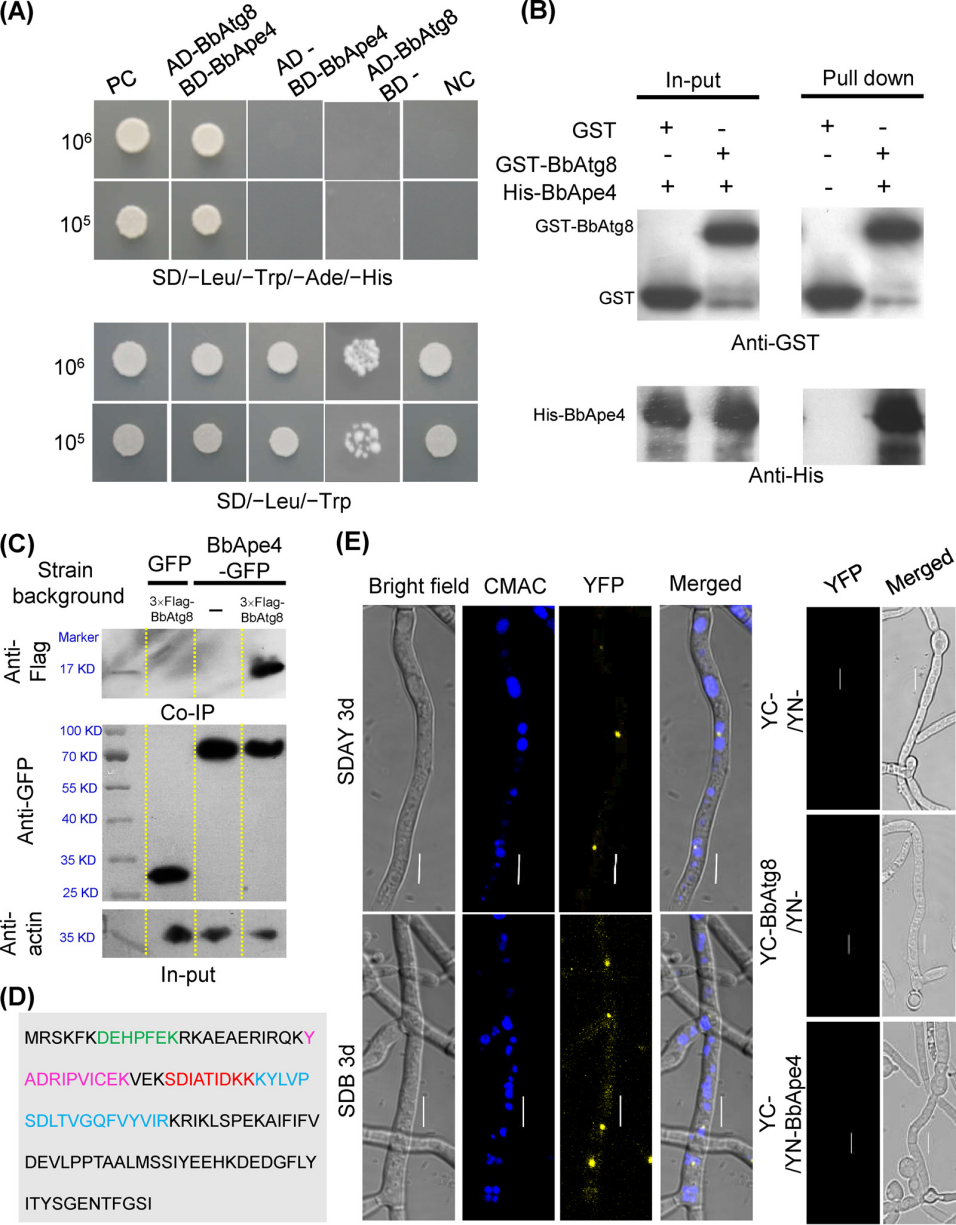
Interaction between BbAtg8 and BbApe4 in Beauveria bassiana. (A) Yeast two-hybrid (Y2H) test. The yeast strain with BbAtg8 and BbApe4 grew well on SD/-Leu-Trp-Ade-His medium, in contrast to the yeast strain with BbApe4. (B) Pulldown assays. GST-BbAtg8 and His-tagged BbApe4 were prepared in a bacterial expression system. The purified GST-BbAtg8 and His-tagged BbApe4 were incubated in an *in vitro* environment. Only GST-BbAtg8 could precipitate the His-tagged BbApe4. (C) Coimmunoprecipitation (co-IP) assays. Three strains with the indicated genetic backgrounds were cultured in SDB. Anti-GFP magnetic beads were then used to precipitate the GFP-tagged proteins in the cell lysate. Only BbApe4-GFP could precipitate proteins that were recognized by the anti-Flag antibody. (D) The precipitated proteins were then characterized by mass spectrometry. Different colors indicate the four identified peptides. (E) BiFC assays for detecting *in vivo* protein interactions. YC-BbAtg8 and YN-BbApe4 were transformed into the wild-type strain, and the resultant transformant was cultured on SDAY/in SDB for 3 days. The fluorescent signals in mycelia were examined using a laser confocal microscope. Fluorescence was not detected in transformants expressing either YC-BbAtg8 or YN-BbApe4 alone or in combination with the respective empty vector. Scale bar = 5 μm.

**FIG 4:**
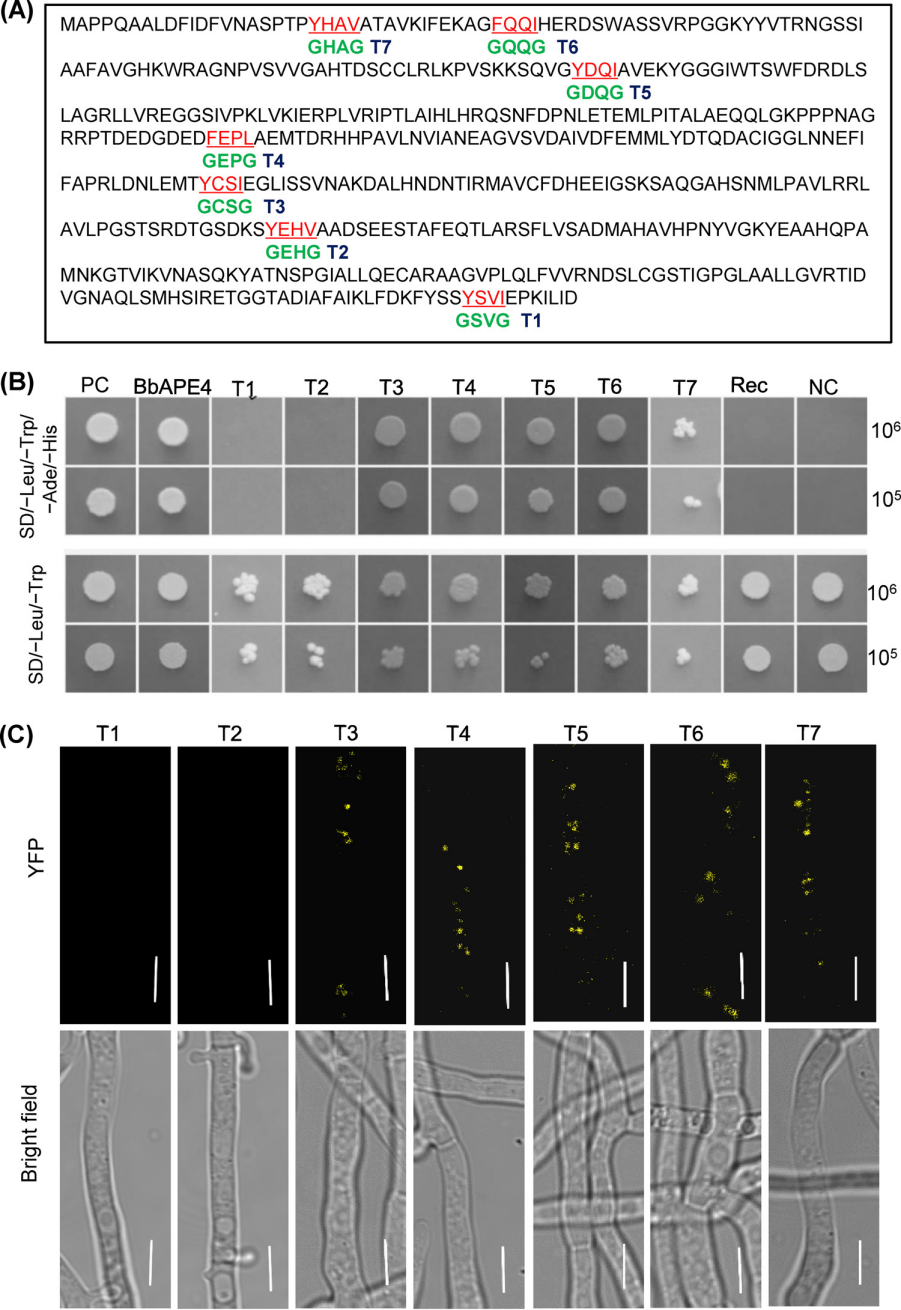
Motifs required for interaction of BbAtg8 with BbApe4. (A) Seven Atg8-interaction motifs (AIM) (T1 to T7) were recognized in BbApe4. (B) Site-directed mutation analyses. AIMs were individually mutated as GXXG, where X is an amino acid residue not mutated. Interaction between BbAtg8 and mutated BbApe4 was determined with a Y2H test. T1 or T2 mutation resulted in no yeast growth on the SD/-Leu-Trp-Ade-His medium. In the Y2H test, positive (PC) and negative (NC) controls worked as expected, and all strains grew well on the SD/-Leu-Trp medium. (C) BiFC assays to assess the interaction of BbAtg8 with different mutated forms of BbApe4. Scale bar = 5 μm.

Among the resultant BbAtg8-interacting proteins, one exhibited high similarity to the yeast aspartyl aminopeptidase (Ape4) (GenBank no. P38821). B. bassiana Ape4 (BbApe4) is a 494-amino acid (aa) protein that is encoded by a 1,561bp open reading frame (ORF) (locus tag BBA_00909) and contains a peptidase_M18 domain (PF02127.15). Yeast two-hybrid (Y2H) assays revealed that only yeast cells containing pGADT7-BbATG8 and pGBKT7-BbAPE4 grew well on a selective medium (Fig. 3A). Pulldown assays indicated that glutathione *S*-transferase (GST)-Atg8 precipitated His-BbApe4 (Fig. 3B). Coimmunoprecipitation (co-IP) assays revealed that BbApe4-GFP precipitated BbAtg8-Flag (Fig. 3C), as confirmed with mass spectroscopy analyses (Fig. 3D; Fig. S5). Bimolecular fluorescence complementation (BiFC) assays (Fig. 3E) revealed weak yellow fluorescent protein (YFP) signals that were observed in the cytoplasm of aerial and submerged mycelia, while stronger signals were present in punctate and globular forms. Seven Atg8-interaction motifs (AIM) (T1 to T7) were identified in BbApe4 (Fig. 4A). Mutation of the T1 or T2 motifs resulted in disrupted interaction between BbApe4 and BbAtg8 (Fig. 4B and C). BLAST and motif search analyses indicated that T1 was observed in Ape4 proteins from both unicellular and filamentous fungi. In contrast, T2 was only present in partially filamentous fungi (Fig. S6A).

10.1128/mbio.03049-22.7 FIG S5 Protein identification. Elutes in the coimmunoprecipitation assay were analyzed with mass spectrometry (MS). MS/MS spectra are shown for four representative peptides of autophagy-related protein 8, and their sequences are shown in the respective spectrum. Download FIG S5, PDF file, 0.2 MB. Copyright © 2023 Ding et al. 2023 Ding et al. https://creativecommons.org/licenses/by/4.0/ This content is distributed under the terms of the Creative Commons Attribution 4.0 International license.

10.1128/mbio.03049-22.8 FIG S6 Bioinformatic and functional assays for Ape4. (A) Bioinformatic analyses of Ape4 proteins in fungal species. Phylogenetic relationships among fungal Ape4 proteins were constructed using neighbor-joining analysis. The bootstrap values at each node from 1,000 replicate tests indicate that there are low sequence similarities. Each protein is followed by the respective fungal species. The Atg8-family interacting motifs (AIM) similar to those (T1 and T2) in B. bassiana are revealed in each protein and framed in red. (B) Complementation of the Saccharomyces cerevisiae Δ*ape4* mutant was accomplished by introducing the *BbAPE4* gene. Yeast resistance to stress was examined on YPDA including various chemicals (final concentration), including ZnCl_2_ (6 mM), NaCl (0.4 M), actinomycin (20 μg/mL), and menadione (0.06 mM), using YPDA as the control. Yeast strains were cultured for 3 days at 30°C. (C) Disruption and complementation of *APE4* in Beauveria bassiana. The partial open reading frame (ORF) is replaced by the *Bar* cassette via homologous recombination. PCR screening of candidate recombination transformants. Lane 1, wild type; lane 2, gene disruption mutant; lane 3, complemented strain; lane M, DNA marker. Southern blot analyses for SalI-digested genomic DNA from the wild-type (lane 1), disruption mutant (lane 2), and complemented strain (lane 3). The electrophoretic positions and sizes of the DNA bands are indicated. (D) Transcriptional analyses of *APE4* were performed in B. bassiana during conidial germination, vegetative growth, and pathogenic growth. (E) BbApe4 trafficking under starvation. The fusion gene *BbAPE4-GFP* was transformed into the wild-type strain. The resultant transformant was grown in Sabouraud dextrose broth, and the obtained mycelia (as control [CK]) were stressed in CZB (CZA without agar) without carbon (–C) or nitrogen (–N) sources. Fluorescent dye CMAC was used to indicate vacuoles. Protein processing of BbApe4 was detected with immunoblotting analyses, using histone 3 (H3) as the control. Lane GFP, a wild-type expressing GFP gene. Bars = 5 μm. (F) Transmission electron microscopy was used to examine autophagy. Fungal strains were cultured in SDB, and the resultant mycelia were stressed under starvation. V, vacuole. Bars = 0.2 μm. (G) Assay for conidial tolerance to heat stress. Conidia were stressed at 42°C. At the indicated time point, conidial viability was examined on germination plates (SPA) at 25°C. (H) Specific analysis of anti-GFP antibody. The strains WT^GFP^, WT, Δ*Bbatg1*, Δ*Bbatg8*, and Δ*Bbatg11* were cultured on SDAY plates for 12 h. The germlings were collected and the proteins were extracted for immunoblotting analyses, using histone 3 (H3) as the control. Download FIG S6, PDF file, 2.2 MB. Copyright © 2023 Ding et al. 2023 Ding et al. https://creativecommons.org/licenses/by/4.0/ This content is distributed under the terms of the Creative Commons Attribution 4.0 International license.

**FIG 5:**
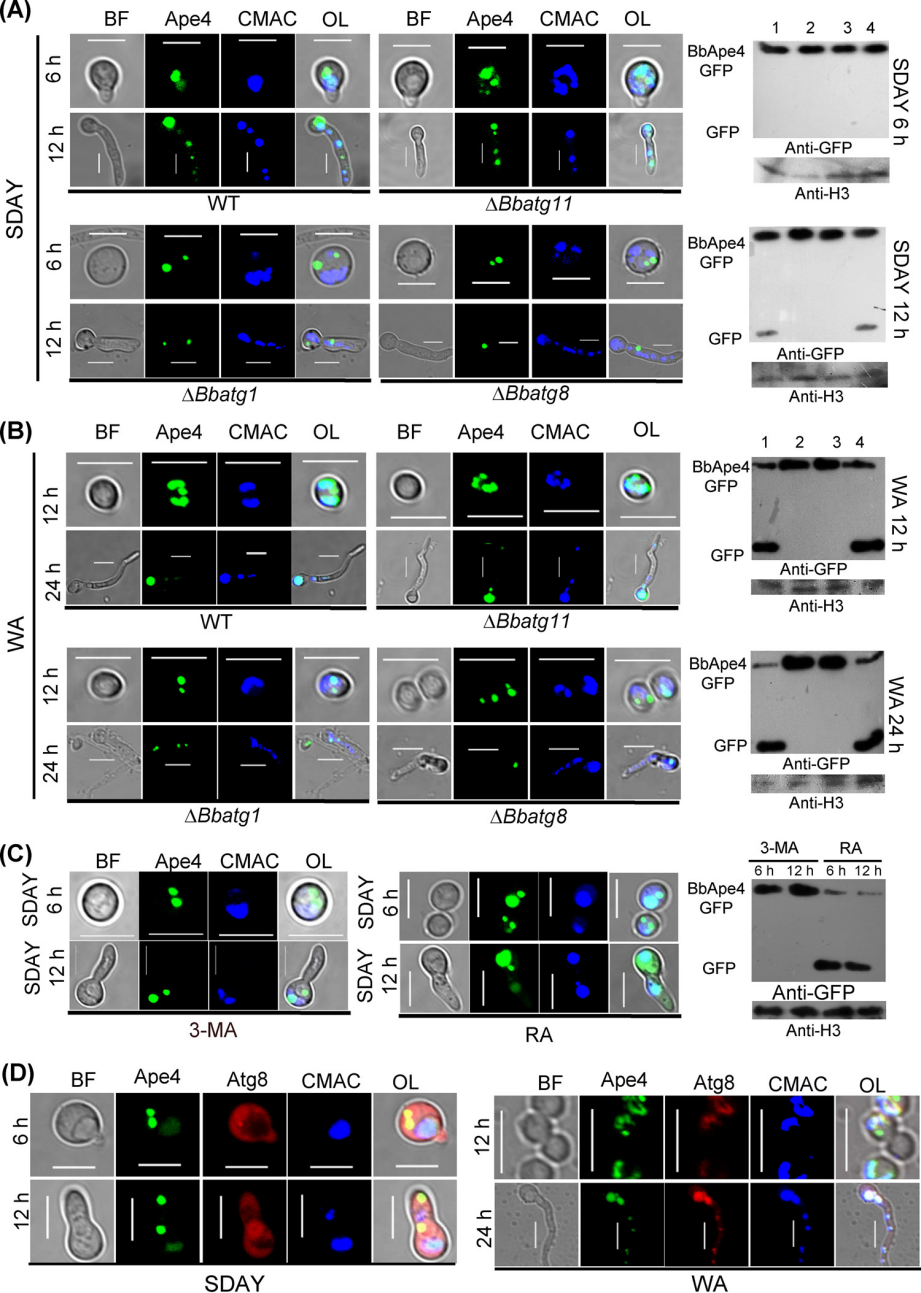
Trafficking of BbApe4 into vacuoles during conidial germination. *BbAPE4* was fused to *GFP*, and the hybrid gene was introduced into the wild-type and Δ*Bbatg1*, Δ*Bbatg8*, and Δ*Bbatg11* mutant strains. Vacuoles were stained with the fluorescent dye CMAC. (A and B) Transformants were inoculated on SDAY (A) and water agarose (WA) (B) plates, followed by cultivation at 25°C. Cells on SDAY plates were sampled at 6 and 12 h, while germlings on WA plates were sampled at 12 and 24 h. Green signals were observed in cells of all fungal types. Green signals appeared in the vacuoles of the Δ*Bbatg11* mutant, similar to that observed for the wild-type strain. However, ablation of *BbATG1* and *BbATG8* blocked the vacuolar targeting of BbApe4. Protein processing of BbApe4-GFP was detected in the wild-type and the Δ*Bbatg1*, Δ*Bbatg8*, and Δ*Bbatg11* mutant strains (lanes 1 through 4, respectively) using Western blot analysis with anti-GFP antibody and histone 3 (H3) as the control. (C) Effects of autophagy inhibitor and activator on the translocation of BbApe4. The wild-type strain with BbApe4-GFP was cultured on an SDAY plate included with the inhibitor 3-methyladenine (3-MA) or the activator rapamycin (RA). Germlings were sampled at 6 and 12 h postincubation. Protein processing of BbApe4-GFP was determined as mentioned above. (D) Colocalization assays. The wild-type strain was transformed with the fusion genes *BbAPE4-GFP* and *mCherry-BbATG8*, with the conidia of the resultant strain cultured on SDAY and WA plates. Fluorescent signals were detected using a laser-scanning confocal microscope. Scale bar = 5 μm.

BbApe4 trafficking during conidial germination was indicated by GFP localization (Fig. 5A). Conidia swelled 6 h postincubation (hpi) on nutrient plates, and GFP globular signals were translocated into the vacuoles of WT and Δ*Bbatg11* mutant strains, with no detection of free GFP. At 12 hpi, GFP signals persisted in the vacuoles of the WT and Δ*Bbatg11* mutant strains, but not in those of the Δ*Bbatg1* and *Bbatg8* mutant strains. Free GFP was detected in the WT and Δ*Bbatg11* mutant strains.

**FIG 6:**
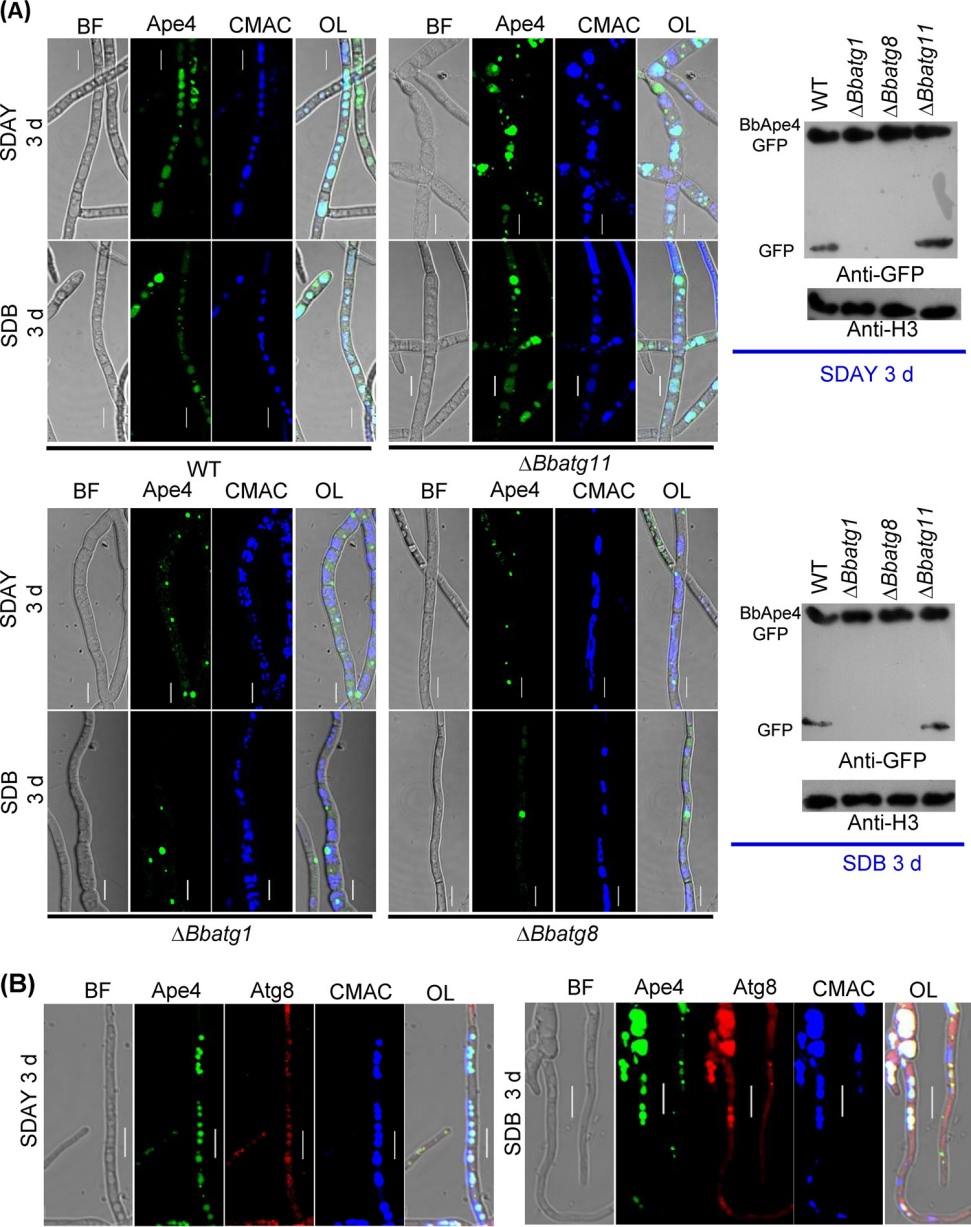
Trafficking of BbApe4 into vacuole in mycelia. (A) *BbAPE4* was fused to *GFP*, and the hybrid gene was introduced into the wild-type and the Δ*Bbatg1*, Δ*Bbatg8*, and Δ*Bbatg11* mutant strains. Vacuoles were stained by the fluorescent dye CMAC. Transformants were inoculated on SDAY/in SDB, and cultured at 25°C for 3 days. Green globular signals were observed in all types of fungal cells. In the Δ*Bbatg11* mutant, green signals appeared in the vacuoles, similar to those in the wild-type strain. Ablation of *BbATG1* and *BbATG8* blocked the vacuolar targeting of BbApe4. In addition, the protein processing of BbApe4 was determined by immunoblotting analyses, using histone 3 (H3) as the control. (B) Colocalization assay. The wild-type strain was transformed with the fusion genes *mCherry-BbATG8* and *BbAPE4-GFP*, and the resultant transformant was stained with the vacuole-specific dye CMAC. Scale bars = 5 μm. BF, bright field; OL, overlapped; d, days.

During starvation conidiation (Fig. 5B), BbApe4 trafficking was similar to that observed under nutrient-rich conditions. Free GFP was detected in the WT and Δ*Bbatg11* mutant strains at both 12 and 24 hpi. Further, 3-MA and RA blocked and enhanced BbApe4 trafficking, respectively (Fig. 5C). Dual-fluorescence assays indicated that BbApe4 and BbAtg8 colocalized during conidial germination in the two above-described conditions (Fig. 5D). These observations persisted in the 3-day-old mycelia (Fig. 6). In control strains that only expressed GFP, GFP signals were distributed throughout their cytoplasm (Fig. S7A). Thus, the presence of BbApe4 in vacuoles is independent of BbAtg11 but dependent on BbAtg1 and BbAtg8.

10.1128/mbio.03049-22.9 FIG S7 Trafficking of Ape4 in B. bassiana. (A) Subcellular localization of green fluorescent protein in mycelia. The gene *GFP* was transformed into the wild type. The resulting transformant was inoculated on SDAY/in SDB and cultured at 25°C. Fungal cells on SDAY plates were sampled at 6 h, 12h, 1 day, 2 days, and 3 days postincubation, whereas the cells in SDB were sampled at 3 days postincubation. Green signals were evenly distributed in cytosol in all types of fungal cells. (B) Vacuolar targeting of BbApe4 with different mutated AIM motifs. BbAPE4 with different single mutation (T3 to T7) was fused with GFP and transformed into the wild type. Mutation of a single motif did not affect the vacuolar targeting of BbApe4. BF, bright field; OL, overlapped. (C) Assay for the interaction of BbApe4 with BbAtg8-β. In the yeast two-hybrid (Y2H) test, the yeast strain with BbAtg8-β and BbApe4 did not grow on the SD/-Leu-Trp-Ade-His medium, in contrast to the yeast for the positive control (PC) and consistent with that of the negative control (NC). The BiFC assay was used to detect the *in vivo* protein interaction. YC-BbATG8-β and YN-BbAPE4 were transformed into the wild-type strain, and the resultant transformant was cultured in SDB for 3 days. The fluorescent signals in mycelia were examined under a laser confocal microscope. No fluorescence was detected in the transformant, which was consistent with the transformant with YC-BbAtg8-β. This result indicated that no interaction is present between BbApe4 and BbAtg8-β. Vacuoles were stained with the vacuole-specific dye CMAC. Scale bars = 5 μm. Download FIG S7, PDF file, 0.6 MB. Copyright © 2023 Ding et al. 2023 Ding et al. https://creativecommons.org/licenses/by/4.0/ This content is distributed under the terms of the Creative Commons Attribution 4.0 International license.

### Phenotypic evaluation of B. bassiana *APE4*.

Ablation of yeast Ape4 resulted in significantly enhanced sensitivities to oxidative stresses caused by menadione (Fig. S6B). *BbAPE4* could suppress the impaired phenotypes in the yeast mutant Δ*ape4* (Fig. S6B). Consequently, disruption and complementation mutants of *BbAPE4* were constructed (Fig. S6C). The conidial yield for the Δ*Bbape4* mutant was 4.31 ± 0.13 × 10^8^ conidia/cm^2^ (mean ± SD), with production reduced by 21% compared to the WT (5.49 ± 0.20 × 10^8^) and complemented (5.11 ± 0.09 × 10^8^) strains. Thus, the ablation of *BbAPE4* resulted in slightly reduced conidial production.

**FIG 7:**
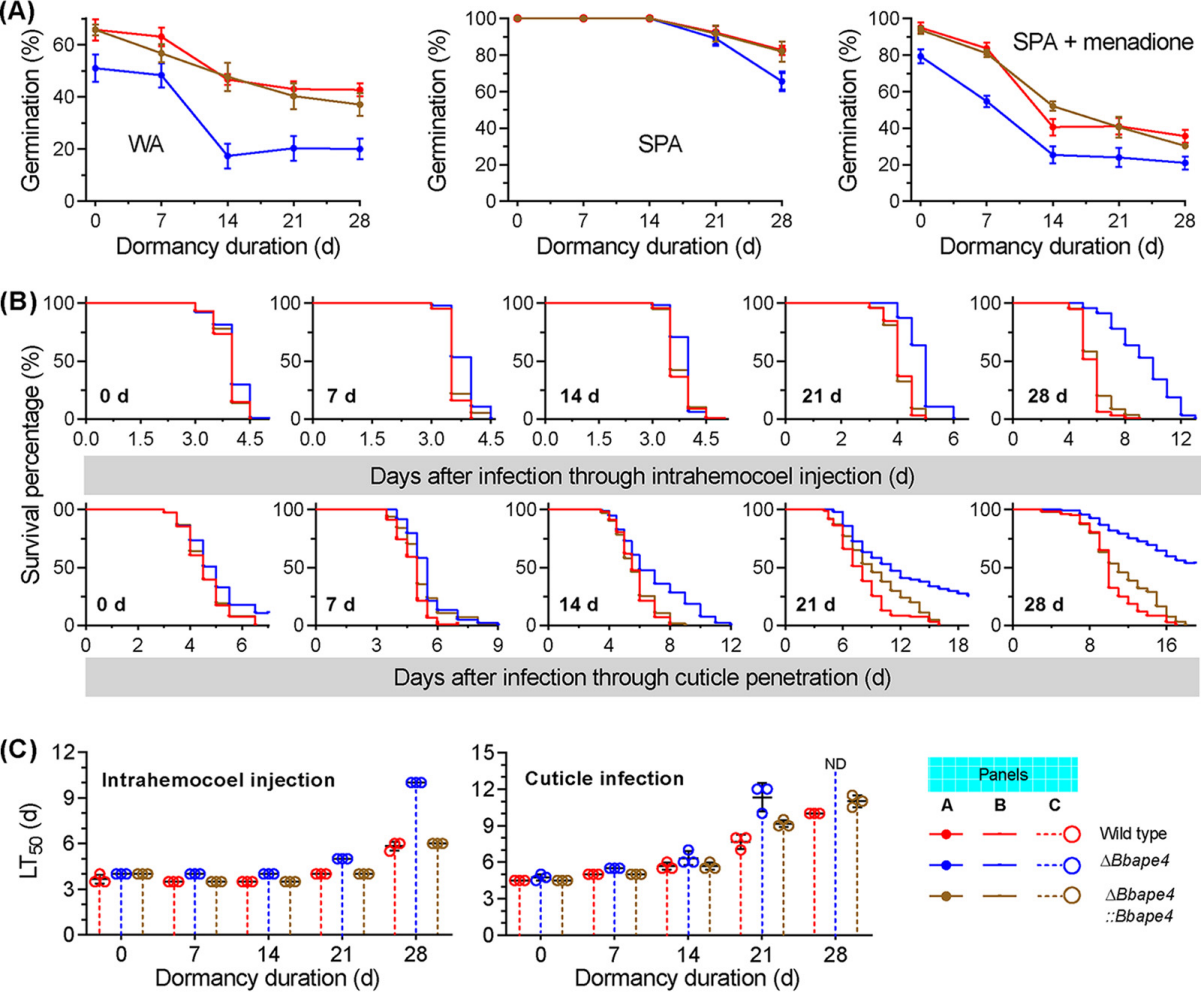
Effects of *BbAPE4* loss on fungal phenotypes. The wild-type, Δ*Bbape4*, and complemented strains were cultured on SDAY plates, and conidia were sampled at the indicated age point. (A to C) Assays for conidial germination (A) and virulence (B and C) were conducted similarly to those in Fig. 1. LT_50_, median lethal time; ND, no LT_50_ value was determined due to mortalities of less than 50%; d, days.

The effects of *BbAPE4* loss on conidial vitality during survival were examined for 4 weeks (Fig. 7A). Newly generated conidia of the Δ*Bbape4* mutant displayed slightly decreased GPs (17.8%) on WA plates compared to that of the WT strain. After 14 DOD, the GPs of Δ*Bbape4* mutant conidia decreased to approximately 25%. On SPA plates, significant differences in GP were not detected between WT and Δ*Bbape4* mutant strains, even after 21 DOD, while only a slight reduction in GP (18.4%) was detected after 28 DOD. However, germination was significantly delayed (Fig. S1C). Under oxidative stress from menadione, the new conidia of the Δ*Bbape4* mutant strain germinated well (GP of ~80%). After 14 DOD, the GP of the gene disruption mutant dramatically decreased to approximately 30% but did not significantly change up to 28 DOD. Images of the germlings grown under the three above-described conditions are shown in Fig. S2C. MTT assays (Fig. S1A) also revealed that the conidial viability of Δ*Bbape4* declined during dormancy, consistent with decreased GPs.

Conidial virulence was assayed either by topical inoculation onto insects or by direct injection into insect hemocoel (Fig. 7B), followed by comparison (Table S1). In the direct injection assay, significant differences were observed between the insect survival curves for the WT and Δ*Bbape4* mutant strains at all sampling time points. However, the two strains could still kill all insects that were assayed. When infecting the hosts through their cuticle, the host survival curves for the WT and Δ*Bbape4* mutant strains were also significantly different across the entire survival period. After 28 DOD, the Δ*Bbape4* mutant did not result in a mortality of >50%. Among the two types of bioassays, the LT_50_ values for the Δ*Bbape4* strain increased with increasing conidial age (Fig. 7C). Notably, the LT_50_ value for the gene disruption mutant in the intrahemocoel injection assay after 28 DOD was 10 days. However, the LT_50_ value could not be determined for the cuticle penetration assay due to its low mortality (<50%).

*BbAPE4* exhibited transcriptional variability during conidial germination, vegetative growth, and pathogenic growth (Fig. S6D). In addition, BbApe4 was translocated into vacuoles during carbon/nitrogen-deficient conditions (Fig. S6E). TEM analyses indicated that BbApe4 loss did not inhibit autophagy (Fig. S6F). In addition, BbApe4 loss did not result in significant phenotypic defects for fungal thermal tolerance (Fig. S6G). Anti-GFP antibody only generated a specific band in the extract from the wild type expressing GFP gene (Fig. S6H).

### Roles of protein motifs in BbApe4 functionality.

**FIG 8:**
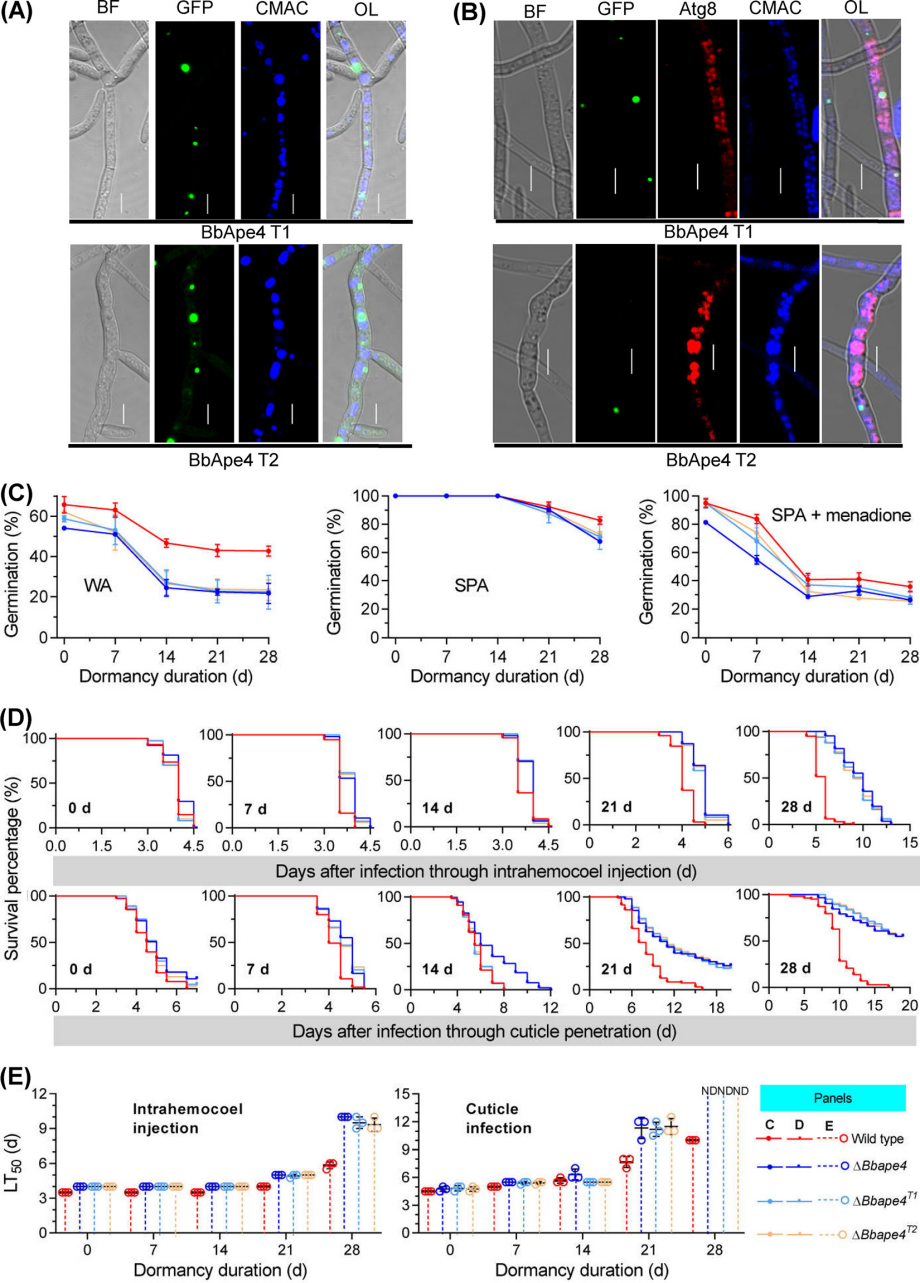
Roles of the T1 and T2 motifs in BbApe4 functionality. *BbAPE4* with a mutation in the T1 or T2 motif was fused with *GFP* and transformed into the wild-type strain to assess protein trafficking and the WT^mChrry-A8^ strain to assess colocalization. (A) Green signals in the mycelia did not translocate into vacuoles, as indicated by staining using the fluorescent dye CMAC. (B) Green signals were not significantly consistent with red signals. Bars = 5 μm. The functionality of mutated BbApe4 was determined in the Δ*Bbape4* mutant strain using the wild-type strain as a positive control. (C to E) Assays for conidial germination (C) and virulence (D and E) were performed as in the experiments shown in Fig. 1. LT_50_, median lethal time; ND, no LT_50_ value was determined due to mortalities of <50%; d, days.

Mutation of the T1 and T2 motifs resulted in defects in vacuolar targeting of the BbApe4 and BbAtg8-BbApe4 interaction (Fig. 8A and B). Additionally, BbApe4 with mutated T1 or T2 could not recover the impaired phenotypes of the Δ*Bbape4* mutant, including conidial germination, response to oxidative stress, and virulence (Fig. 8C to E). In contrast, mutation of the T3 to T7 motifs did not affect vacuolar targeting of BbApe4 (Fig. S7B), and these mutated forms of BbApe4 could recover the defective phenotypes of Δ*Bbape4* mutants (Fig. S3D to F). Thus, the T1 and T2 motifs are required for BbApe4 vacuolar targeting and functionality.

### Vacuolar targeting of BbApe4 is dependent on autophagic roles of BbAtg8.

The tripeptide at the C terminus of BbAtg8 is also essential for B. bassiana autophagy. TEM microscopy (Fig. S8A) revealed that ablation of *BbATG8* led to the absence of autophagy and that BbAtg8^T^ did not recover autophagy compared to the wild type. In addition, the contribution of BbAtg8 to autophagosome formation was dependent on the tripeptide at the extreme C terminus (Fig. S8B). The tripeptide functionality in selective autophagy was examined in three sets of transformants (Fig. S8C). BbAtg8^T^ could not recover the translocation of fluorescent signals from the cytoplasm into vacuoles in the Δ*Bbatg8* mutant. Thus, the tripeptide of BbAtg8 is indispensable for pexophagy, mitophagy, and the cytoplasm-to-vacuole transport (Cvt) pathway.

10.1128/mbio.03049-22.10 FIG S8 Tripeptide at the extreme carboxyl-terminus is indispensable for the autophagy role of BbAtg8. The truncated *BbATG8* (*BbATG8^T^*) was transformed into the Δ*Bbatg8* mutant strain, and the generated strain was named Δ*Bbatg8^A8T^*. (A) Representative images of autophagic bodies. Under a transmission electronic microscope, autophagic bodies (yellow arrow) were detected in the vacuole of the wild-type strain and not in the Δ*Bbatg8* and Δ*Bbatg8^A8T^* strains. Scale bars = 0.2 μm. (B) Fluorescent view of the autophagic flux. Fused genes of *GFP-BbATG8* and *GFP-BbATG8^T^* were integrated into the wild-type strain. Green punctate signals (white arrow) were seen in the transformant with *GFP-BbATG8* and not in the transformant with *GFP-BbATG8^T^*. Scale bars = 5 μm. (C) Detecting pexophagy, mitophagy, and the Cvt pathway. Peroxisome, mitochondrion, and BbApeI were indicated with GFP. For pexophagy and mitophagy, fungal strains were cultured in SDB for 2 days, and the resulting mycelia were starved for 3 h. Scale bars = 10 μm. For the Cvt pathway, the strains were cultured on SDAY plates for 12 h. Scale bars = 5 μm. In the wild-type strain, green signals (pink arrow) were translocated into vacuoles. However, no significant green fluorescence was observed in the vacuoles of the Δ*Bbatg8* and Δ*Bbatg8^A8T^* strains. BF, bright field; OL, overlapped. Download FIG S8, PDF file, 0.6 MB. Copyright © 2023 Ding et al. 2023 Ding et al. https://creativecommons.org/licenses/by/4.0/ This content is distributed under the terms of the Creative Commons Attribution 4.0 International license.

**FIG 9:**
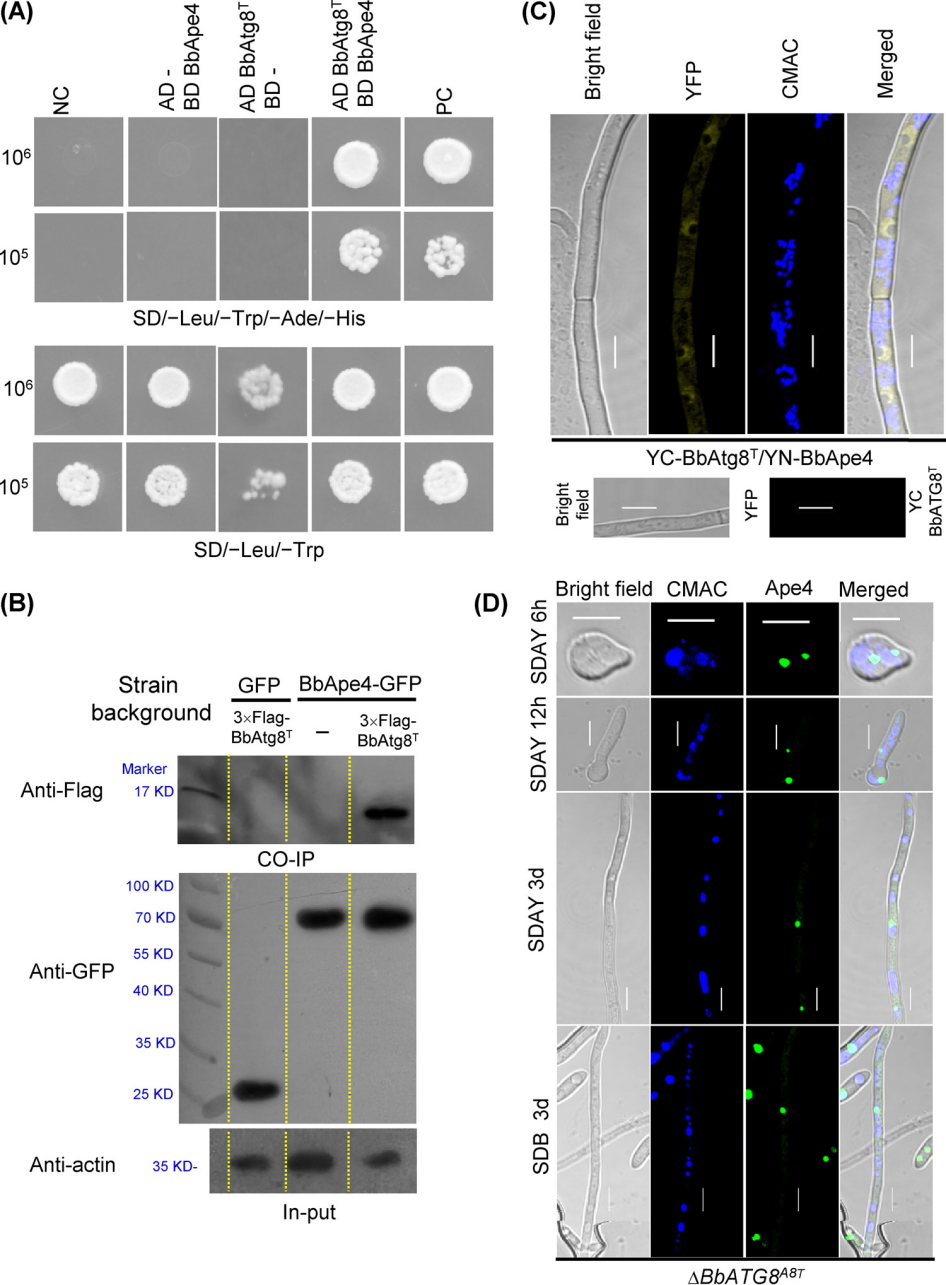
The tripeptide at the extreme carboxyl terminus of BbAtg8 is necessary for BbApe4 translocation. (A) Y2H test results. The yeast strain with BbAtg8^A8T^/BbApe4 grew well on SD/-Leu-Trp-Ade-His medium, indicating that truncation of the tripeptide at the extreme carboxyl-terminus of BbAtg8 did not influence its interaction with BbApe4. (B and C) Co-IP (B) and BiFC (C) assays were used to assess *in vivo* interactions of BbApe4 with BbAtg8^T^. (D) Translocation of BbApe4 to vacuoles. The hybrid gene *BbAPE4-GFP* was transformed into the Δ*Bbatg8^A8T^* mutant strain. Transformants were then cultured on SDAY plates or in SDB. Samples were taken from SDAY plate cultures at 6 h, 12 h, and 3 days. Mycelia from cultures grown in SDB were collected at 3 days. Vacuoles are indicated by staining with the fluorescent dye CMAC. Green punctate signals were detected in all samples and did not translocate into vacuoles. d, days. Scale bar = 5 μm.

As described above (Fig. 3), vacuolar targeting of BbApe4 was mediated through its direct interaction with BbAtg8. Protein interaction assays indicated that truncation of the tripeptide at the C terminus did not impair the interaction of BbAtg8 with BbApe4 (Fig. 9A to C) but blocked the translocation of BbApe4 into vacuoles (Fig. 9D). Direct interaction was not observed between BbApe4 and BbAtg8-β (the alternative splicing isoform of BbAtg8) (Fig. S7C). Thus, the autophagic role of BbAtg8 mediates vacuolar targeting of BbApe4.

### Autophagic effects of BbAtg8 are essential for conidial persistence.

**FIG 10:**
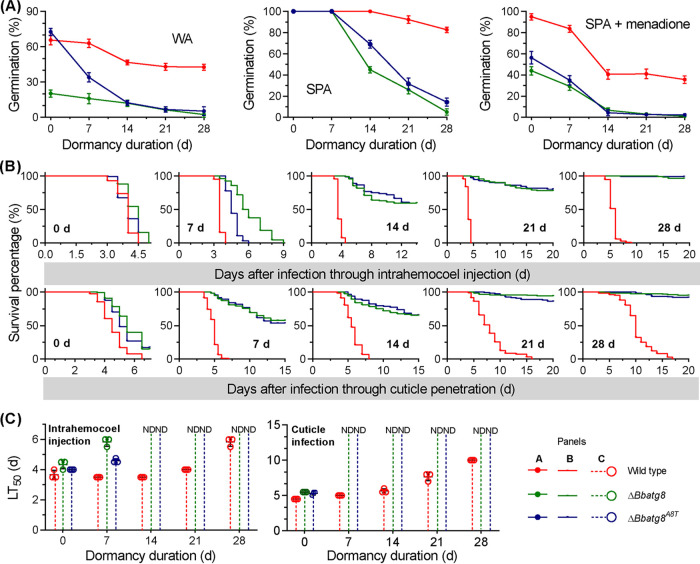
Effects of tripeptides at the extreme carboxyl-terminus on the biological functions of BbAtg8. BbAtg8 was mutated to lack the tripeptide at the extreme carboxyl terminus. The truncated *BbATG8* (*BbATG8*^T^) was then introduced into the Δ*Bbatg8* mutant strain, and the resultant strain was termed Δ*Bbatg8^A8T^*. (A to C) Phenotypic assessments for conidial germination (A) and virulence (B and C) were conducted as in the experiments shown in Fig. 1. LT_50_, median lethal time; ND, no LT_50_ value was determined due to mortalities of <50%; d, days.

The conidial life span and viability during survival were examined across 4 weeks of dormancy (Fig. 10A). A significant difference in conidial GP was not observed between the WT and Δ*Bbatg8*^A8T^strain conidia at the initial time of dormancy on WA plates. As dormancy time increased, the GP of the Δ*Bbatg8*^A8T^ strain conidia dramatically decreased, becoming similar to that of the Δ*Bbatg8* mutant strain conidia after 14 DOD. In addition, significant differences in GP trends were not observed between the Δ*Bbatg8*^A8T^ and Δ*Bbatg8* mutant strain conidia on SPA plates. Similarly, significant differences in GP trends were not observed between the two strains under oxidative stress. Thus, the Δ*Bbatg8*^A8T^ strain exhibits survival characteristics like those of the Δ*Bbatg8* strain, except for the germination of newly formed conidia under starvation. However, the germination trend was significantly delayed (Fig. S1D). Germling morphologies grown under the three above-described conditions are shown in Fig. S2D.

The conidial virulence of the Δ*Bbatg8*^A8T^ strain was evaluated using two bioassays, followed by a comparison of results for the WT and Δ*Bbatg8* mutant strains (Fig. 10B). In the injection assay, the host survival curves for the Δ*Bbatg8*^A8T^ and Δ*Bbatg8* strains were significantly different up to seven DOD, but not after longer incubation times (Table S1). In the cuticle infection assay, host survival trends for the Δ*Bbatg8*^A8T^ and Δ*Bbatg8* strains slightly differed after 21 DOD (*P = *0.04), but their cumulative mortalities were similar. Overall, the host survival curves for the Δ*Bbatg8*^A8T^ and Δ*Bbatg8* strains were not significantly different at any other time point, except at the aforementioned point. The LT_50_ value trends of the Δ*Bbatg8*^A8T^ and Δ*Bbatg8* strains within the two bioassays were similar across the entire dormancy period (Fig. 10C), except for the injection assay of conidia that were dormant for 7 days. These results demonstrate that the carboxyl-terminal tripeptide is critical for conidial viability.

Overexpression of *BbATG8* and truncated *BbATG8* (*BbATG8^T^*) in the WT strain resulted in distinct influences on conidial viability and virulence. Strain WT^A8^ exhibited slightly increased GPs on WA plates. As dormancy time increased, the GP of the WT^A8^ strain dramatically decreased after 14 DOD on the three medium types, which was similar to that observed in the Δ*Bbatg8* mutant strain. The WT^A8T^ strain exhibited a slight decrease in GP during dormancy on WA plates, but no significant difference in conidial GP was observed between the WT and WT^A8T^ strains on SPA and menadione plates (Fig. S3G). Germling morphologies are shown in Fig. S2E.

In the injection bioassay, the WT^A8T^ strain exhibited a similar host survival trend as the WT strain, while the WT^A8^ strain exhibited significantly delayed host survival trends. In the cuticle penetration bioassay, the WT^A8T^ strain exhibited a slightly delayed host survival trend compared to that of the WT strain. The host survival trend for the WT^A8^ strain was significantly prolonged, similar to that of the Δ*Bbatg8* mutant strain (Fig. S3H). LT_50_ values for all bioassays are shown in Fig. S3I. Overall, overexpression of *BbATG8* significantly impaired conidial persistence and virulence.

## DISCUSSION

Autophagy and different ATG genes have been associated with the aging process in vegetative cells from multiple eukaryotes (18). Conidia from filamentous fungi (a form of dormant cells) enter a dormant state after maturation on spore-generating structures (19). In this study, autophagy was shown to be essential for the persistence of conidia after maturation, with B. bassiana conidia used as a model.

The chronological life span (CLS) is the length of time that nongrowing cells remain viable, with viability representing the cell’s ability to recover mitosis (20). Fungal spores develop both endogenous and exogenous dormancy to survive in environments for longer periods, with a conidial exit from exogenous dormancy triggered by water or exogenous nutrient availability (21). B. bassiana conidia accumulate abundant nutrients (22, 23), although only some conidia can germinate under starvation conditions. In addition, exogenous nutrients can recover conidial germination capacity (15, 24). Thus, the germinating capacity under nutrient-rich and nutrient-limited conditions indicates conidial viability and vitality, respectively. Spore longevity after maturation differs among fungal species (25). Autophagy is essential for the conidial life span in B. bassiana, and the autophagy activity is finely regulated. Atg1 and Atg8 are hub regulators of autophagy and play similar roles. In filamentous fungi (e.g., Podospora anserina), autophagy acts as an antiaging mechanism in vegetative cells and determines mycelial longevity (10). The results of this study are consistent with this conserved role in determining the longevity of filamentous fungi, regardless of cell type. Conidial aging is an oxidation-dependent process in N. crassa. Antioxidant enzymes (e.g., catalase and superoxide dismutase) and metabolites (e.g., ergothioneine) are required for conidial viability during dormancy (5, 6). However, B. bassiana conidia lose tolerance to oxidative stress during long-term dormancy, which is associated with autophagy. Pexophagy has been previously shown to be involved in B. bassiana resistance to oxidative stress (15), reinforcing that autophagy functions as a critical mechanism for removing damaged organelles and proteins due to oxidative stress (26). In addition, autophagy-related signaling regulates cellular antioxidant responses (27) and BbAtg8 maintains intracellular superoxide dismutase activity (14). These results indicate that autophagy and ATGs together affect the conidial antioxidant system (e.g., cellular mechanisms and enzymes) that is involved in aging during dormancy.

Virulence is a determinant of the biocontrol efficacy of entomopathogenic fungi (28). Conidial germination is a critical step in the propagation and infectivity of B. bassiana (23). The ability of B. bassiana conidia to germinate under nutrient-starvation conditions decreased with prolonged storage, wherein survival trends considerably decreased 2 weeks postmaturation. These results reflect that conidia are dormant cells with very low metabolic activities (29). B. bassiana autophagy contributes to the mobilization of endogenous nutrient reserves to promote conidial germination under oligotrophic conditions (14, 15). A similar mechanism is conserved among filamentous fungi (e.g., Aspergillus oryzae and Sordaria macrospora) (30, 31). Among the core ATG genes, *ATG1* and *ATG8* are needed for autophagosome induction and expansion, respectively. *ATG11* encodes a scaffold protein that is needed to achieve selective autophagy (7). Entomopathogenic fungi (e.g., B. bassiana) encounter reactive oxygen species produced by host immune defenses (e.g., O_2_^−^) when infecting host hemocoel (32). Importantly, autophagy contributes to conidial resistance to oxidative stress during aging. Considering that pathogen virulence results from combined host-pathogen interactions (33), autophagy is critical for fungal virulence during survival due to its role in maintaining conidial vitality under oligotrophic and oxidative stress exposure conditions. Three ATG genes (*BbATG1*, *BbATG8*, and *BbATG11*) in B. bassiana contribute differently to conidial survival, indicating that the total autophagic flux is essential for conidial survival within the environment, while selective autophagy is relatively less important.

The autophagic degradation of proteinaceous components relies on proteolytic enzymes in vacuoles (16, 34). The aspartyl aminopeptidase (Ape4) of filamentous fungi belongs to the M18 family of metallopeptidases and cleaves peptides and proteins from the N-terminal regions of proteins (35). B. bassiana aspartyl aminopeptidase (BbApe4) does not play a significant role in maintaining conidial viability across the entire dormancy period but is responsible for conidial vitality under oligotrophic and oxidative stress exposure conditions, thereby ultimately contributing to conidial virulence. These results indicate that BbApe4 significantly contributes to conidial aging and postmaturation infective potential. Moreover, this study describes the first functional Ape4 identified in filamentous fungi. Ape4 in baker’s yeast significantly contributes to cellular resistance to oxidative (menadione) stresses. BbApe4 is an ortholog of yeast Ape4 in B. bassiana. In the opportunistic human pathogen yeast Cryptococcus neoformans, Ape4 is required for autophagy and cellular resistance to stresses (e.g., thermal and osmotic stresses), which determines the ability of the fungus to survive hostile environments within hosts (36). Oxidative stress can be induced by numerous environmental stresses (e.g., high temperature, radiation, and chemicals) (37) and host immune defenses (38). Importantly, autophagy acts as a cleaner of damaged macromolecules and organelles (39). Although the *in vivo* substrates of Ape4 are not known, it may contribute to protein degradation during stress, because it is a vacuolar protease. These results suggest that Ape4 is an autophagy-related proteinase that is important for conidial persistence and recovery from dormancy.

Ape4 in Saccharomyces cerevisiae is synthesized as a cytoplasmic protein, and only a small portion of Ape4 is translocated into vacuoles under normal growth conditions, although trafficking of Ape4 into vacuoles is enhanced by starvation stress (40). BbApe4 was observed to translocate into vacuoles during conidial germination and hyphal growth during nutritive conditions. The processing of BbApe4 is dependent on autophagy and occurs in vacuoles, similar that that observed for yeast Ape4 (40, 41). Nevertheless, the two fungi have developed different mechanisms for the vacuolar transport of Ape4. Ape4 binds to Atg19 in yeast, and the resulting complex is transported into vacuoles via the cytoplasm with the vacuole targeting (Cvt) pathway (40), in which Atg11 and Atg19 act as scaffold proteins and receptors, respectively (42). Specifically, the receptor sequentially binds to Atg11 and Atg8, allowing the movement of materials into the phagophore assembly site (PAS) (42). In addition, Atg8 anchors on the growing phagophore and mediates membrane tethering and expansion during autophagosome formation (43). However, the vacuolar importation of Ape4 in B. bassiana is not dependent on Atg11-mediated selective autophagy but, rather, is accomplished via direct interaction with Atg8, which is defined as a simplified Cvt pathway (sCvt pathway). The autophagic role of Atg8 is indispensable for vacuolar targeting of BbApe4 in B. bassiana. The Ape4-Atg8 interaction occurs during conidial germination and hyphal development, requiring a more efficient supply of nutrients and energy (14, 44). These results indicate that BbApe4 translocates into the autophagosome without a receptor, which might allow more efficient hydrolase transportation for autophagic degradation. The results indicate that the other Atg8-related activities are involved in conidial germination under oligotrophic conditions 0 and 7 days after dormancy (Fig. 10A). Thus, the Atg8-dependent targeting route for vacuolar hydrolase is essential for conidial recovery from a long-term dormancy. The Atg8-family interacting motif (AIM) plays essential roles in the interactions between Atg8 and autophagic receptors, including, for example, Atg19 (yeast) and p62/sequestosome 1 (human) (45). Two AIMs in BbApe4 are localized at the C terminus and are necessary for binding with BbAtg8. Notably, the AIM site (YXXI) at the extreme end of the C terminus is conserved in fungal Ape4 homologs, including in yeast and filamentous fungi. This suggests that this simplified pathway for selective autophagy might be prevalent in diverse fungi.

Taken together, these results suggest that autophagy confers environmental persistence for dormant filamentous fungal cells (e.g., conidia) by controlling the turnover of endogenous nutrients and cellular aging. A novel trafficking route for hydrolase translocation from the cytoplasm into the vacuole during conidial germination and growth was identified in this study. Moreover, the results presented here highlight the critical roles of autophagy in the environmental persistence of dormant cells and improves our understanding of the diverse mechanisms involved in selective autophagy.

## MATERIALS AND METHODS

### Microbial strains and culture media.

The wild-type B. bassiana ARSEF2860 (WT) strain and derived strains are shown in Table S2A. Fungal strains were routinely maintained on Sabouraud dextrose agar (SDAY, containing 4% glucose, 1% peptone, 1.5% agar, and 1% yeast extract), as previously described (46). The yeast strains YH109 and BY4741 were cultured in yeast extract-peptone-dextrose (YPD, containing 1% yeast extract, 2% peptone, and 2% glucose), and their associated transformants were cultured on a synthetic defined medium with requisite amino acids (TaKaRa, Dalian, China).

10.1128/mbio.03049-22.2 TABLE S2 Experimental materials used in this study. (A) Bacterial and fungal strains. (B) Primers for various purposes. Download Table S2, DOCX file, 0.03 MB. Copyright © 2023 Ding et al. 2023 Ding et al. https://creativecommons.org/licenses/by/4.0/ This content is distributed under the terms of the Creative Commons Attribution 4.0 International license.

### Assays for the persistence of conidial viability and vitality.

The above-mentioned strains were cultured on SDAY plates for 7 days at 25°C. Conidia were then harvested from the plate surfaces and stored at 25°C. Conidial germination, oxidative stress tolerance, and virulence were determined during the subsequent 4 weeks, as previously described (15). Each assay was repeated in triplicate.

**(i) Conidial germination.** To assess conidial germination, WA plates (1.5% agarose) were used as the oligotrophic medium, while SPA plates (2% sucrose, 0.5% peptone, and 1.5% agar) were used as a rich medium to determine conidial viability. To examine the conidial response to oxidative stress, conidial suspensions were inoculated on SPA plates supplemented with 0.06 mM menadione. Inoculations on SPA plates without modifications were used as controls.

**(ii) Virulence assay.** Fungal virulence was tested against Galleria mellonella larvae via cuticle penetration and intrahemocoel injection. The survival trends of hosts over time were recorded and plotted as Kaplan-Meier curves. Statistical differences between paired curves were analyzed using log-rank tests.

**(iii) MTT assay.** Conidial viability was evaluated by MTT assays, as previously described (47). Briefly, conidia (1 × 10^7^) were suspended in phosphate-buffered saline (PBS) solution, and MTT solution was added to a final concentration of 5 mg/mL. Conidial suspensions were then incubated in the dark at 37°C for 3 h. The suspensions were centrifuged, and then dimethyl sulfoxide was added to the precipitates to dissolve formazan, followed by measurement of the optical density at 579 nm (OD_579_) values of the supernatants.

### Assays for autophagy processes during conidial germination.

Autophagy processes were observed as previously described (48). Specifically, the fusion gene *GFP-BbATG8* was transformed into the wild-type strain. The resultant strain was cultured on WA and SPA plates, followed by sampling of mycelia at 6, 12, and 24 h postincubation. After staining with 7-amino-4-chloromethylcoumarin (CMAC), fluorescence was observed with a fluorescence microscope. BbAtg8 lipidation was evaluated in germlings on SPA and WA plates, followed by detection with immunoblotting using a monoclonal anti-flag antibody. Transmission electron microscopy (TEM) was also used to determine autophagy processes in conidia and germlings. The effects of the autophagy inhibitor (3-methyladenine [3-MA]) and activator (rapamycin [RA]) on autophagic processes were also examined. Further, the presence of BbAtg8 in conidia (100 mg) was also determined through Western blot analyses.

### Protein interaction of BbAtg8 with BbApe4.

To assess BbAtg8 and BbApe4 interactions, yeast two-hybrid (Y2H) assays were conducted according to the manufacturer’s instructions using a Matchmaker GAL4 two-hybrid system 3 kit (Clontech Laboratories, California, USA). Briefly, cDNA was reverse transcribed from mRNA of 2-day-old submerged mycelia in Sabouraud dextrose broth (SDB) medium and then ligated into the pGADT7 vector to generate a cDNA library of pGADT7-LS. The *BbATG8* coding sequence was amplified with primers P1 and P2 (Table S2B) and then cloned into pGBKT7 to generate the plasmid pGBKT7-Atg8. The plasmid and library plasmids were then transformed into yeast YH109. One putative interaction protein was identified as B. bassiana Ape4 (locus tag BBA_00909). *BbAPE4* was then amplified with the primer pair P3/P4 to examine the interaction between BbAtg8 and BbApe4.

Seven motifs (W/F/Y-X-X-L/I/V) (44) were predicted in BbApe4 and subsequently mutated into G-X-X-G by PCR with the primers P7 to P30 (Table S2B). The wild-type and mutated forms of *BbAPE4* were cloned into pGBKT7, generating pGBKT7-X (where X indicates different forms of *BbAPE4*). *BbATG8* was also cloned into pGADT7, and the resultant plasmid was paired with the plasmid pGBKT7-X and transformed into a yeast strain.

Pulldown assays were conducted with the recombinant proteins GST, GST-BbAtg8, and BbApe4-His prepared by transforming Escherichia coli strain BL21 with pGEX 4T-3, pGEX 4T-3-BbAtg8, and pGEX 32a-BbApe4, respectively. Protein preparation and purification were performed as previously described (48). The GST-BbAtg8 and His-tagged BbApe4 were purified and incubated in an *in vitro* environment. GST or GST-BbAtg8 was used as prey to capture BbApe4-His protein, and the presence of BbApe4-His in the eluent was detected by immunoblotting using a monoclonal anti-His antibody. Coimmunoprecipitation (co-IP) assays were then used to verify the interaction of BbApe4 with BbAtg8. The fusion gene BbAPE4-GFP and *GFP* were transformed into the wild-type strain, generating the WT^Ape4-Gfp^ and WT^Gfp^ strains, respectively. *BbATG8* was then fused to the 3×Flag gene and transformed into the WT^Ape4-Gfp^ and WT^Gfp^ strains. To precipitate Ape4-Gfp, the protein solution was incubated with 10 μL of anti-GFP magnetic beads (P2132, Beyotime Biotechnology, Shanghai, China). After washing, the presence of BbAtg8 in the eluent was detected by immunoblotting using a monoclonal anti-Flag antibody (F7425, Sigma) and confirmed with mass-spectrum analyses (48). Bimolecular fluorescence complementation (BiFC) assays were used to examine protein interactions in B. bassiana, as previously described (48). Specifically, the coding sequence for *BbATG8* was cloned into p0380T-YC-MB, generating the plasmid p0380T-YC-ATG8-B. *BbAPE4* was also ligated into p0380TM-YN-S, generating the plasmid p0380T-APE4-YN-S. The paired plasmids were then transformed into the wild-type strain. The transformants were cultured in SDB, and fluorescent signals were observed with a fluorescence microscope (LSM 710, Carl Zeiss Microscopy GmbH, Jena, Germany). Plasmids containing split YFP genes were used as negative controls. All primers used for PCR amplifications are shown in Table S2B.

### Analysis of the BbApe4 trafficking route from the cytoplasm into vacuoles.

To evaluate the trafficking route of BbApe4, the p0380-BbApe4-GFP-sur vector was introduced into the wild-type, Δ*Bbatg1*, Δ*Bbatg8*, and Δ*Bbatg11* strains (14, 15). In addition, the fusion gene *mCherry-BbATG8* was integrated into the wild type. The resultant strain (WT^CA8^) was transformed with p0380-BbApe4-GFP-sur to observe colocalization. Conidia of each strain were then cultured on WA and SDAY plates. Mycelia on SDAY plates were then sampled at 6 and 12 h postincubation (hpi), while fungal cells on WA plates were sampled at 12 and 24 hpi. In SPB medium (SPA without agar), mycelia were cultured for 3 days. After staining with CMAC, mycelial fluorescence was observed with a fluorescence microscope. Protein processing of BbApe4 was then detected by immunoblotting using a monoclonal anti-GFP antibody.

### Functional analyses of *BbAPE4*.

Yeast genetic manipulations were conducted as previously described (49). Specifically, the fused DNA fragment with the *Kan^R^* cassette was used to replace the *APE4* ORF in the yeast strain BY4741, generating the Δ*ape4* strain. *BbAPE4* was then introduced into the locus site of the *APE4* ORF in the Δ*ape4* strain, generating the Δ*ape4*::*Bbape4* strain. Cellular resistance to stress was subsequently examined on YPDA (YPD medium plus agar) supplemented with ZnCl_2_ (6 mM), NaCl (0.4 M), actinomycin (20 μg/mL), or menadione (0.06 mM), followed by incubation of plates for 3 days at 30°C.

Targeted gene disruption of *BbAPE4* and complementation of gene loss were conducted by homologous recombination and ectopic insertion, respectively, as previously described (15). Briefly, the flanking sequences of the gene were amplified with the primer pairs P47/P48 and P49/P50 (Table S2B). To achieve gene complementation, the entire gene was amplified with the primer pair P51/P52. The transformants were then screened with PCR using the primer pair P53/P54 (Table S2B) and further confirmed by Southern blot analysis with a DIG DNA labeling and detection kit (Roche, Germany). DNA fragments amplified with the primer pair P55/P56 (Table S2B) were used as templates for probe preparation.

In addition, to validate the importance of motifs in BbApe4 trafficking and functionality, the wild-type and mutated forms of *BbAPE4* were cloned into pGBKT7, generating pGBKT7-X (where X indicates different forms of BbAPE4). The resultant plasmids were transformed into the wild-type strain for trafficking assays and the Δ*Bbape4* strain for functional assays. Protein trafficking and autophagosome ultrastructure assays were performed using the previously mentioned methods. Phenotypic assays were also conducted as described above.

### Truncation analysis of BbAtg8.

Truncated *BbATG8* (*BbATG8^T^*) was prepared by PCR and then introduced into the Δ*Bbatg8* mutant strain, followed by evaluation of its expression with Western blot analysis. The resultant strain was termed Δ*Bbatg8^A8T^*.

Autophagosome ultrastructures were examined with TEM (24), while autophagic flux was measured with the fluorescent reporter method (15). Specifically, the coding sequences of BbAtg8 and BbAtg8^T^ were amplified and then cloned into vectors to confer chlorsulfuron resistance (50). The hybrid gene was transformed into the Δ*Bbatg8* strain, and the functioning of BbAtg8^T^ in selective autophagy was determined by analyzing pexophagy and mitophagy, as previously described (15). In addition, the *TEF1* promoter and *trpC* terminator were amplified and cloned into the plasmid p0380-ptrA (48), generating the plasmid p0380-TEF-MCS-TER-ptrA (pTMTP). To label mitochondria and peroxisomes, the primer pairs P83/P84 and P85/P86 were used to amplify gene fragments, and the resultant fragments were cloned into the pTMTP plasmid. Mycelia were concomitantly obtained from SDB medium and subjected to starvation. Pexophagy and mitophagy were then detected using a fluorescence microscope.

The interaction between BbAtg8^T^ and BbApe4 was examined as described above. In addition, the translocation of BbApe4 into the vacuole was analyzed in the Δ*Bbatg8^A8T^* strain. The fusion gene *BbAPE4-GFP* was cloned into the pTMTP plasmid, and the resultant plasmid was introduced into the Δ*Bbatg8^A8T^* strain. Assays for protein translocation and interaction were then performed as described above. Overexpression of *BbATG8* and *BbATG8^T^* was accomplished by transforming the wild-type strain with each gene. The resultant strains were confirmed by Western blot analysis and termed WT^A8^ or WT^A8T^, respectively. Phenotypic assays were conducted as described above. All strains and primers that were used are listed in Table S2B.

### qRT-PCR assays.

Transcriptional profiles of *BbAPE4*, *BbATG1*, *BbATG8*, and *BbATG11* were determined during conidial germination, fungal growth, conidial maturation, and host infection, as previously described (51). The relative transcript levels of each gene were calculated for paired comparisons using the fungal actin gene as an internal reference and the 2^–ΔΔ^^*C*_T_^ method for quantification (52). All primers used for reverse transcription-quantitative PCR (qRT-PCR) assays are shown in Table S2B.
