# Supplementary material for: The Entomopathogenic Fungus Beauveria bassiana Employs Autophagy as a Persistence and Recovery Mechanism during Conidial Dormancy
Source: mBio. 2023 Feb 21;14(2):e03049-22. doi: 10.1128/mbio.03049-22 (PMC10128008; doi:10.1128/mbio.03049-22)

Fig. S1 Analyses of conidial viability. (A) Fungal strains were cultured on SDAY plates for 7 d till conidiation, and conidia were stored at 0, 7, 14, 21, and 28 d. The sampled conidia were stained with 3-(4,5-dimethylthiazol-2-yl)-2,5-diphenyltetrazolium bromide (MTT). The resultant MTT-formazan was dissolved in dimethyl sulfoxide, and the absorbance of solution was examined at 579 nm. Fungal strains were cultured on SDAY plates for 7 d till conidiation, and conidia were stored at 0, 7, 14, 21, and 28 d. The sampled conidia were inoculated on nutrient-rich plates (SPA) and incubated at 25°C. Conidial germination was determined every two hours for 24 h and indicated as germination percentage (%). (B) the wild-type and autophagy-null strains; (C) the wild-type, Δ*Bbape4* and its complementation mutant strains; (D) the wild-type, Δ*Bbatg8* and Δ*Bbatg8*^{A8T} strains.

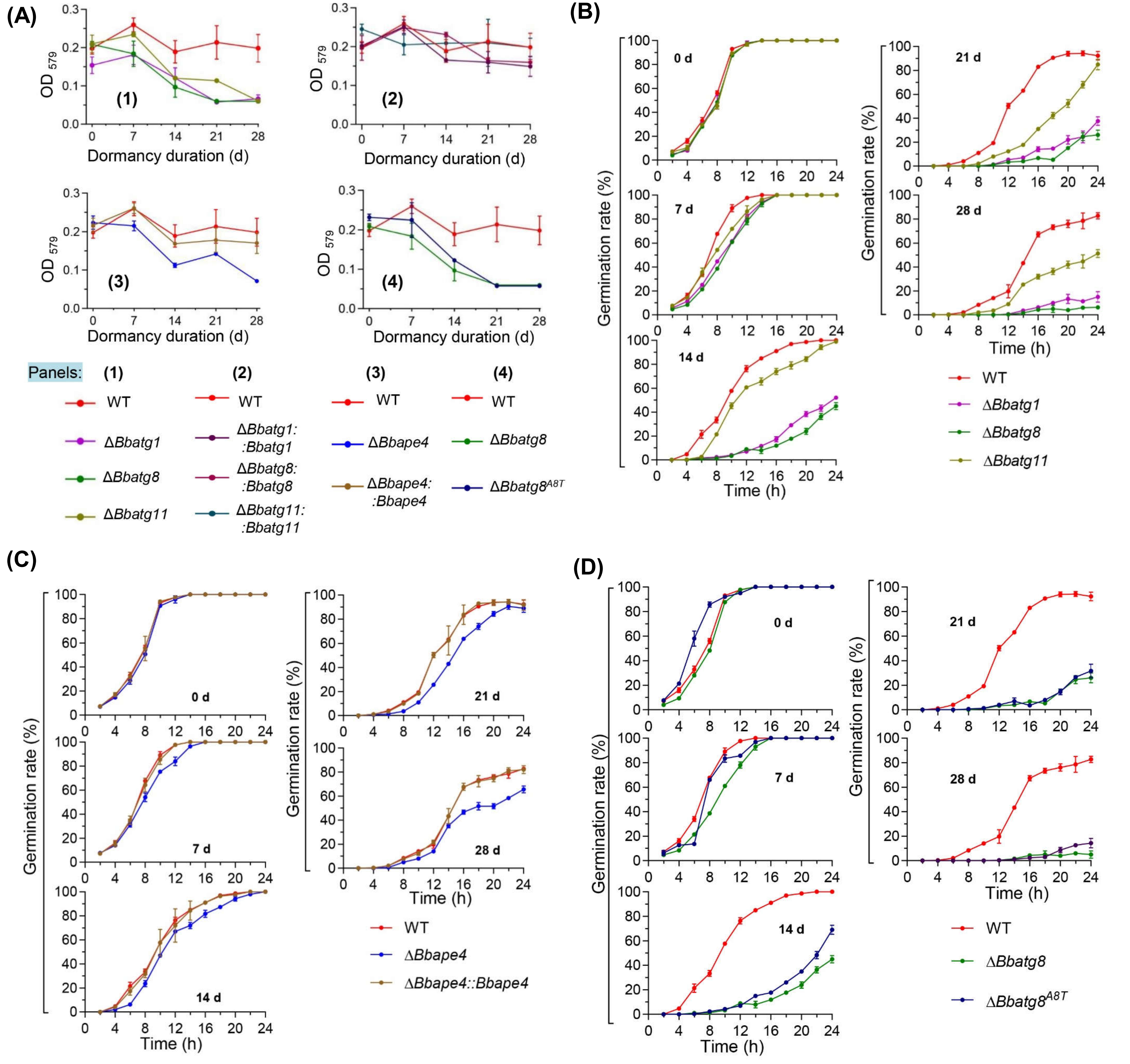

Supplement: FIG S1 [file mbio.03049-22-s0003.pdf]
