# Supplementary material for: The Entomopathogenic Fungus Beauveria bassiana Employs Autophagy as a Persistence and Recovery Mechanism during Conidial Dormancy
Source: mBio. 2023 Feb 21;14(2):e03049-22. doi: 10.1128/mbio.03049-22 (PMC10128008; doi:10.1128/mbio.03049-22)

Fig. S2 Microscopic view of conidial germination. Fungal strains were cultured on SDAY plates for 7 d till conidiation, and conidia were stored at 0, 7, 14, 21, and 28 d. The sampled conidia were inoculated on water agar plates (WA), nutrient-rich plates (SPA), and stress plates (SPA + menadione). After an incubation of 24 h at 25°C, morphologies of fungal cells were recorded. Bars: 10 μm. (A) the wild-type, Δ*Bbatg1*, Δ*Bbatg8*, Δ*Bbatg11* mutant strains; (B) the wild-type and complementation mutants for Δ*Bbatg1*, Δ*Bbatg8*, Δ*Bbatg11* mutant strains; (C) the wild-type, Δ*Bbape4* and its complementation mutant strains; (D) the wild-type , Δ*Bbatg8* and Δ*Bbatg8*^{A8T} strains; (E) *BbATG8* and truncated *BbATG8* (*BbATG8*^{T}) was transformed into the wild-type strain (WT), and the resultant strains were named as WT^{A8} and WT^{A8T}, respectively.

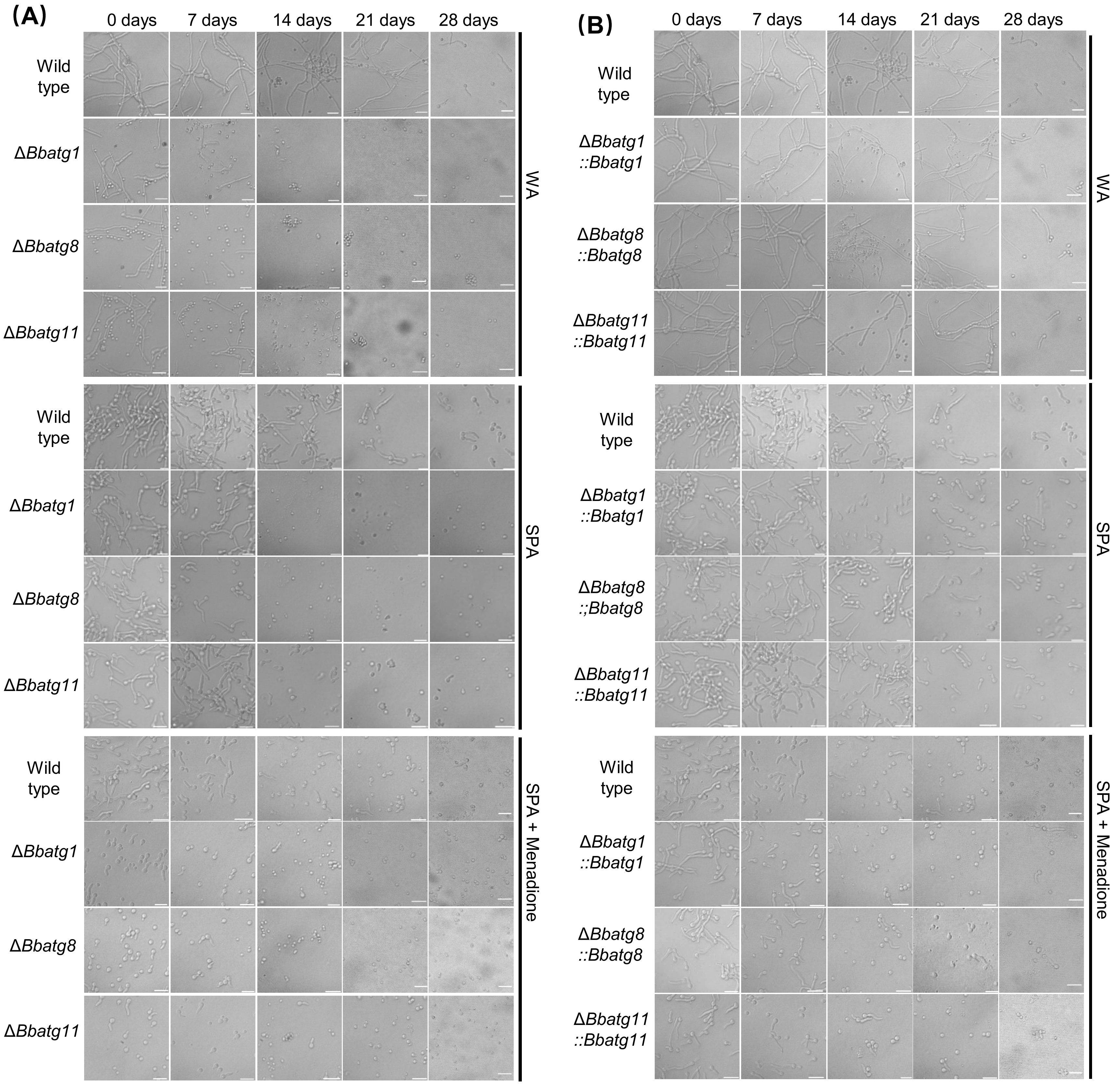

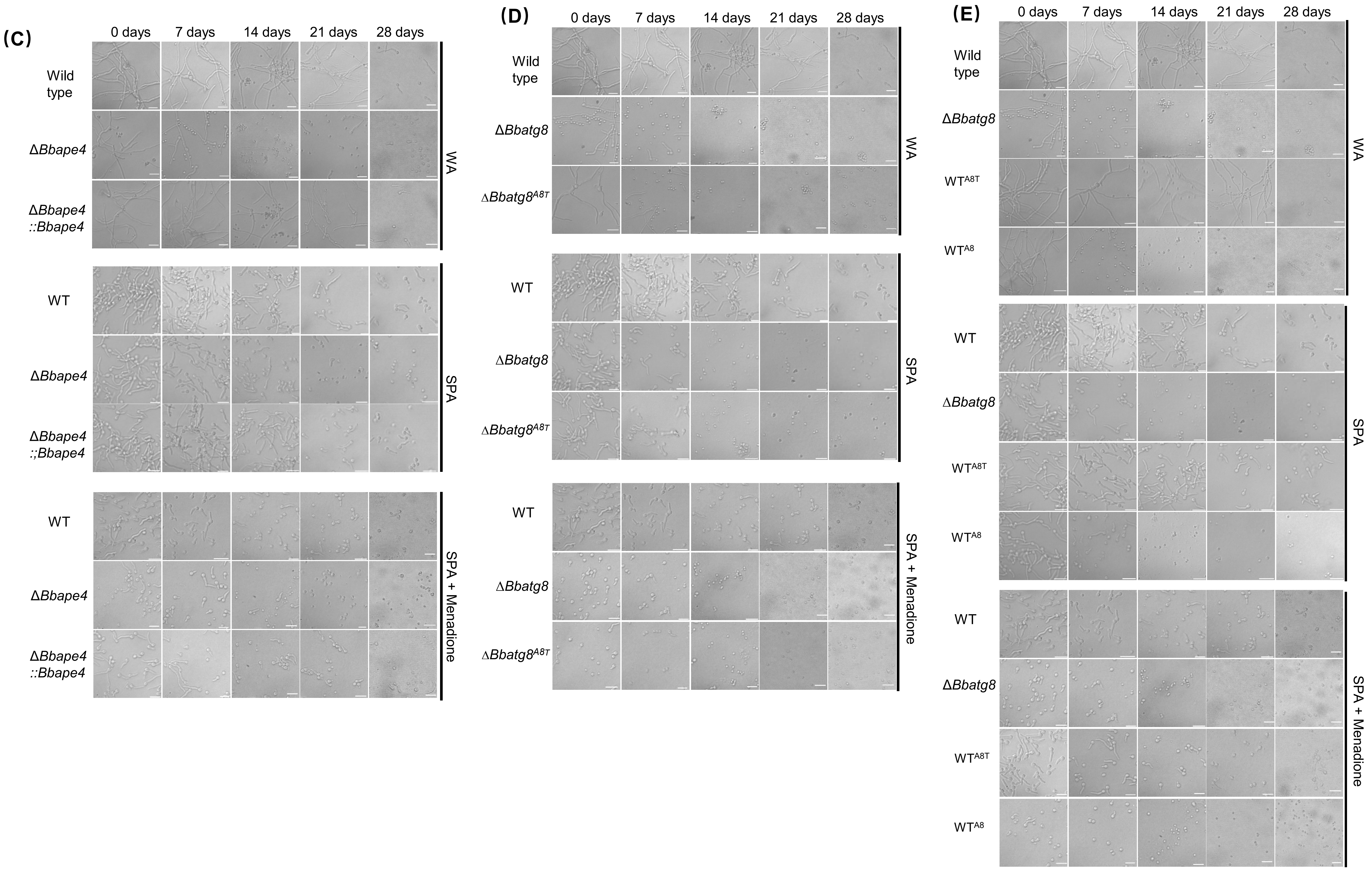

Supplement: FIG S2 [file mbio.03049-22-s0004.pdf]
