# Supplementary material for: The Entomopathogenic Fungus Beauveria bassiana Employs Autophagy as a Persistence and Recovery Mechanism during Conidial Dormancy
Source: mBio. 2023 Feb 21;14(2):e03049-22. doi: 10.1128/mbio.03049-22 (PMC10128008; doi:10.1128/mbio.03049-22)
Supplement: TABLE S1 [file mbio.03049-22-s0001.docx]

**Table S1 *P*-values from Log-rank test for the paired survival curves in bioassay.**

| **Paired comparison** | **Intrahemocoel injection assay** | | | | | **Cuticle infection assay** | | | | |
| --- | --- | --- | --- | --- | --- | --- | --- | --- | --- | --- |
|  | **0 ^*^** | **7** | **14** | **21** | **28** | **0 *** | **7** | **14** | **21** | **28** |
| Bioassay for the wild-type (WT), Δ*Bbatg1*, Δ*Bbatg8* and Δ*Bbatg11* strains | | | | | | | | | | |
| WT/Δ*Bbatg1* | <0.0001 | <0.0001 | <0.0001 | <0.0001 | <0.0001 | <0.0001 | <0.0001 | <0.0001 | <0.0001 | <0.0001 |
| WT/Δ*Bbatg8* | <0.0001 | <0.0001 | <0.0001 | <0.0001 | <0.0001 | <0.0001 | <0.0001 | <0.0001 | <0.0001 | <0.0001 |
| WT/Δ*Bbatg11* | 0.5586 | <0.0001 | <0.0001 | <0.0001 | <0.0001 | <0.0001 | <0.0001 | <0.0001 | <0.0001 | <0.0001 |
| Δ*Bbatg1*/Δ*Bbatg8* | 0.5492 | 0.0013 | <0.0001 | <0.0001 | 0.1427 | 0.8074 | 0.3401 | 0.0347 | 0.0102 | 0.7834 |
| Δ*Bbatg1*/Δ*Bbatg11* | <0.0001 | <0.0001 | <0.0001 | <0.0001 | <0.0001 | 0.7178 | <0.0001 | <0.0001 | <0.0001 | <0.0001 |
| Δ*Bbatg8*/Δ*Bbatg11* | <0.0001 | <0.0001 | <0.0001 | <0.0001 | <0.0001 | 0.8897 | <0.0001 | <0.0001 | <0.0001 | <0.0001 |
| Bioassay for the WT and Δ*Bbape4* strains | | | | | | | | | | |
| WT/Δ*Bbape4* | 0.0145 | <0.0001 | 0.0004 | <0.0001 | <0.0001 | 0.0013 | <0.0001 | <0.0001 | <0.0001 | <0.0001 |
| Bioassay for the WT, Δ*Bbatg8* and Δ*Bbatg8^A8T^*strains | | | | | | | | | | |
| WT/Δ*Bbatg8* | <0.0001 | <0.0001 | <0.0001 | <0.0001 | <0.0001 | <0.0001 | <0.0001 | <0.0001 | <0.0001 | <0.0001 |
| WT/Δ*Bbatg8 ^A8T^* | 0.0179 | <0.0001 | <0.0001 | <0.0001 | <0.0001 | <0.0001 | <0.0001 | <0.0001 | <0.0001 | <0.0001 |
| Δ*Bbatg8*/Δ*Bbatg8^A8T^* | <0.0001 | <0.0001 | 0.7542 | 0.7035 | 0.1692 | 0.1719 | 0.6533 | 0.7493 | 0.0434 | 0.3232 |

*: day(s) of dormancy.
