# Supplementary material for: The Entomopathogenic Fungus Beauveria bassiana Employs Autophagy as a Persistence and Recovery Mechanism during Conidial Dormancy
Source: mBio. 2023 Feb 21;14(2):e03049-22. doi: 10.1128/mbio.03049-22 (PMC10128008; doi:10.1128/mbio.03049-22)

Fig. S4 Autophagy homeostasis is critical for conidial germination. (A) Transcriptional analyses of autophagy-related gene (*ATG*) 1, 8 and 11 were performed in *B. bassiana* during conidial germination, vegetative growth, and pathogenic growth. (B) Conidia germination were examined on water agar plates (WA), nutrient-rich plates (SPA), and stress plates [SPA + menadione (M)]. Effects of autophagy inhibitor and activator on conidial germination were conducted by adding 3-methyladenine (3-MA) and rapamycin (RA) into the indicated medium, respectively. Germination percentage was examined at 24 h post incubation, and morphologies of germinating cells were recorded (C). Bars: 10 μm. (D) The transgenic strain expressing *GFP-BbATG8* was cultured on SDAY plates included with 3-MA or RA. Fluorescent dye CMAC and GFP-BbAtg8 was used to indicate vacuole and autophagic flux, respectively. Lipidation of BbAtg8 was detected with immunoblotting analyses, using histone 3 (H3) as control. The wild type was used to determine the specificity of anti-Flag antibody. Bars: 5 μm.

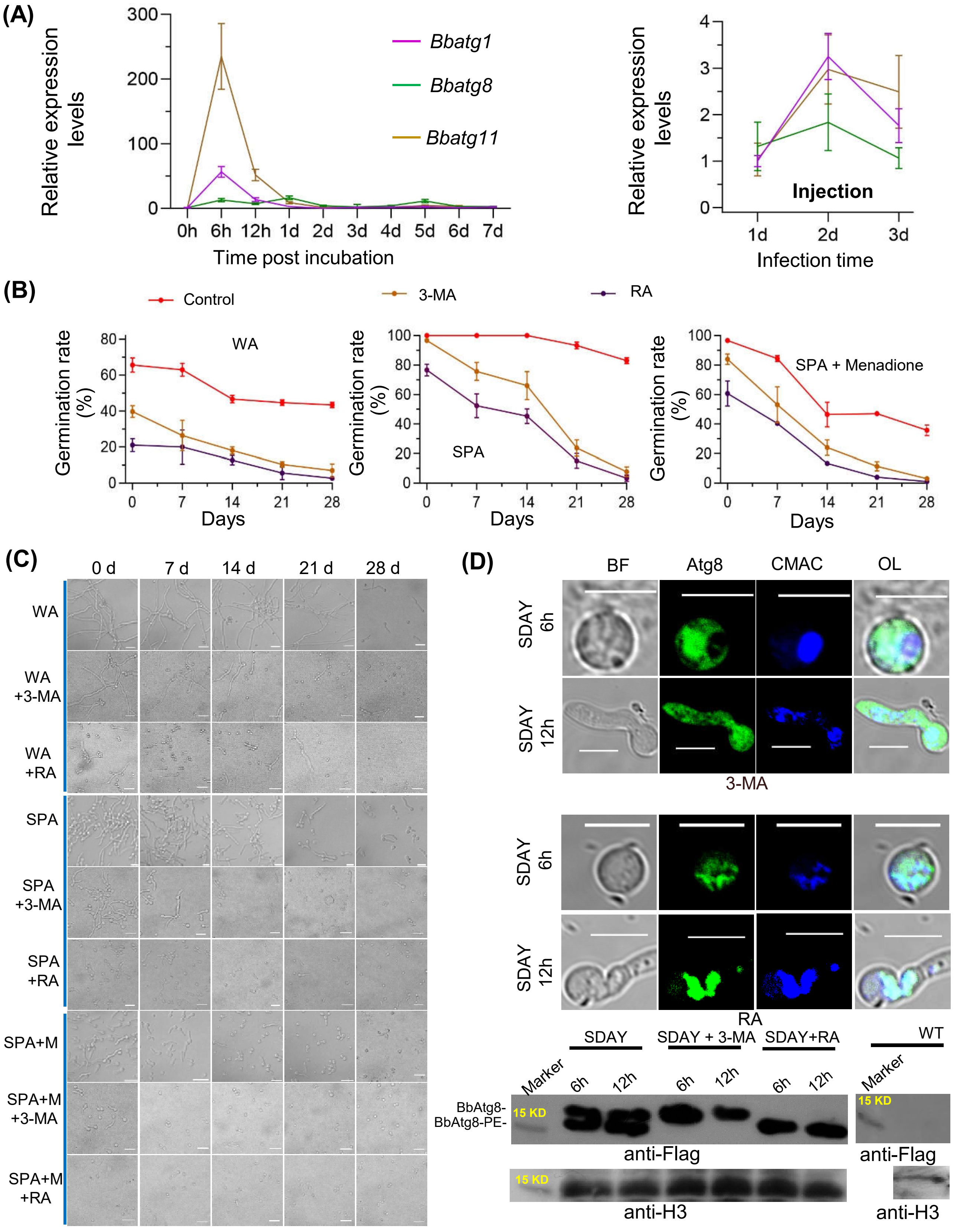

Supplement: FIG S4 [file mbio.03049-22-s0006.pdf]
