# Supplementary material for: The Entomopathogenic Fungus Beauveria bassiana Employs Autophagy as a Persistence and Recovery Mechanism during Conidial Dormancy
Source: mBio. 2023 Feb 21;14(2):e03049-22. doi: 10.1128/mbio.03049-22 (PMC10128008; doi:10.1128/mbio.03049-22)

Fig. S5 Protein identification. Elutes in co-immunoprecipitation assay were analyzed with mass spectrometry (MS). MS/MS spectra are shown for four representative peptides of autophagy-related protein 8, and their sequences are shown in the respective spectrum.

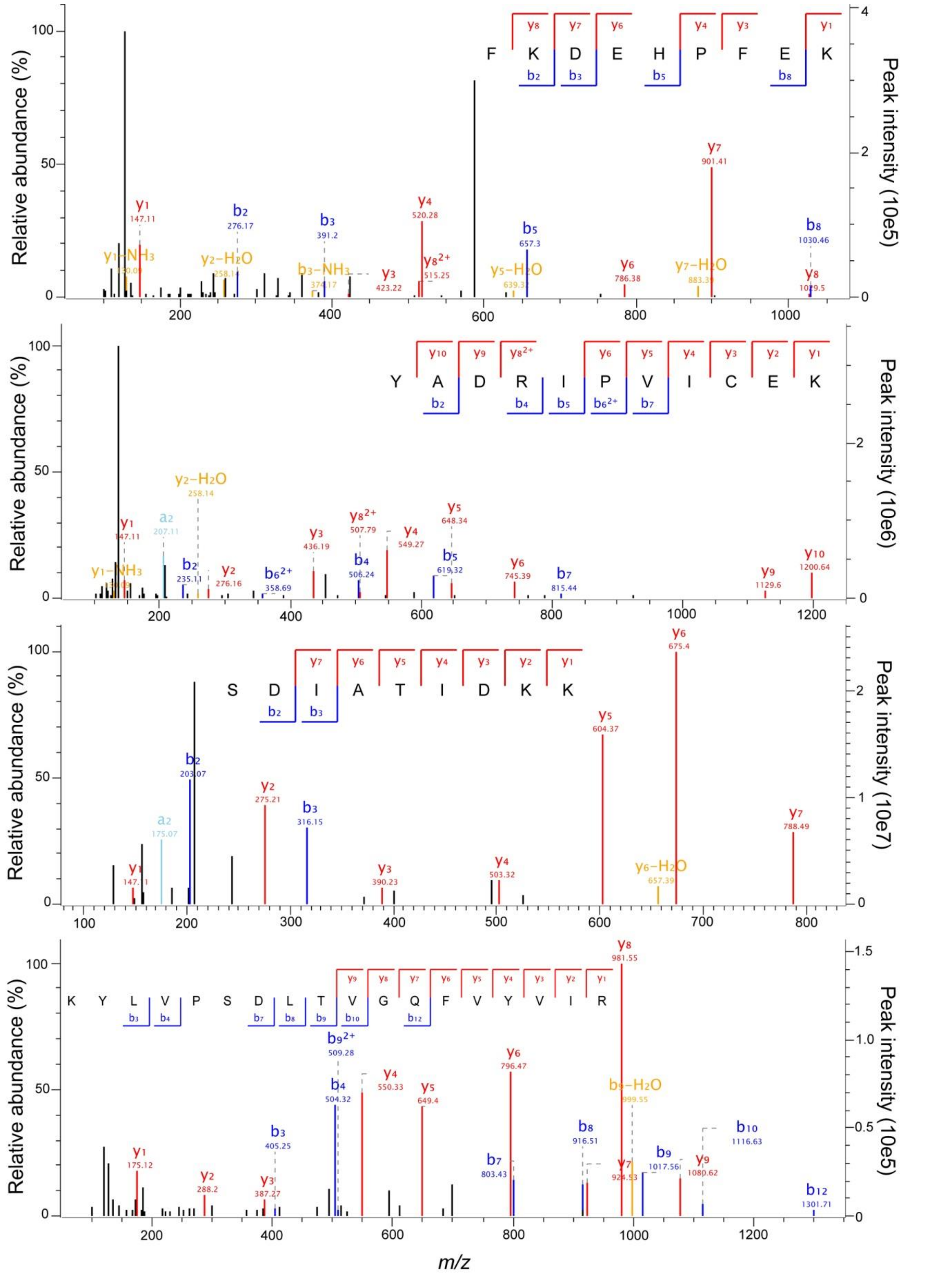

Supplement: FIG S5 [file mbio.03049-22-s0007.pdf]
