# Supplementary material for: The Entomopathogenic Fungus Beauveria bassiana Employs Autophagy as a Persistence and Recovery Mechanism during Conidial Dormancy
Source: mBio. 2023 Feb 21;14(2):e03049-22. doi: 10.1128/mbio.03049-22 (PMC10128008; doi:10.1128/mbio.03049-22)

Fig. S6 Functional complementation assay. (A) Bioinformatic analyses of Ape4 proteins in fungal species. Phylogenetic relationships among fungal Ape4 proteins (GenBank accession number) were constructed by Neighbor-Joining analysis. The bootstrap values at each node from 1000 replicate tests indicate that there are low sequence similarities. Each protein is followed by respective fungal species. The Atg8-family interacting motifs (AIM) similar with those (T1 and T2) in *B. bassiana* are revealed in each protein and framed in red. (B) Complementation of the *Saccharomyces cerevisiae* Δ*ape4* mutant was accomplished by introducing *BbAPE4* gene. Yeast resistance to stress was examined on YPDA included various chemicals (final concentration), including $ZnCl_2$ (6 mM), NaCl (0.4 M), actinomycin (20 μg/ml), and menadione (0.06 mM), using YPDA as control. Yeast strains were cultured for 3 d at 30°C. (C) Disruption and complementation of *APE4* in Beauveria bassiana. (A) Partial open reading frame (ORF) is replaced by Bar cassette via homologous recombination. (B) PCR screening of candidate recombination transformants. Lane 1: wild type; lane 2: gene disruption mutant; lane 3: complemented strain and lane M: DNA marker. Southern blot analyses for *Sal*I-digested genomic DNA from the wild-type (lane1), disruption mutant (lane2) and complemented strain (lane 3). The electrophoretic positions and sizes of the DNA bands are indicated. (D) Transcriptional analyses of APE4 were performed in B. bassiana during conidial germination, vegetative growth, and pathogenic growth. (E) BbApe4 trafficking under starvation. The fusion gene BbAPE4-GFP was transformed into the wild-type strain. The resultant transformant was grown in Sabouraud dextrose broth, and the obtained mycelia (as control (CK)) were stressed in CZB (CZA without agar) without carbon (-C) or nitrogen (-N) source. Fluorescent dye CMAC was used to indicate vacuoles. Protein processing of BbApe4 was detected with immunoblotting analyses, using histone 3 (H3) as control. Lane GFP: a wild-type expressing GFP gene. Bars: 5 μm. (F) Transmission electron microscopy was used to examine autophagy. Fungal strains were cultured in SDB, and the resultant mycelia were stressed under starvation. "V": vacuole. Bars: 0.2 μm. (G) Assay for conidial tolerance to heat stress. Conidia were stressed under 42°C. At the indicated time point, conidia viability was examined on germination plates (SPA) at 25°C. (H) Specific analysis of anti-GFP antibody. The strains WT^{GFP}, WT, Δ*Bbatg1*, Δ*Bbatg8* and Δ*Bbatg11* were cultured on SDAY plates 12 h. The germlings were collected and the protein were extracted for immunoblotting analyses, using histone 3 (H3) as control.

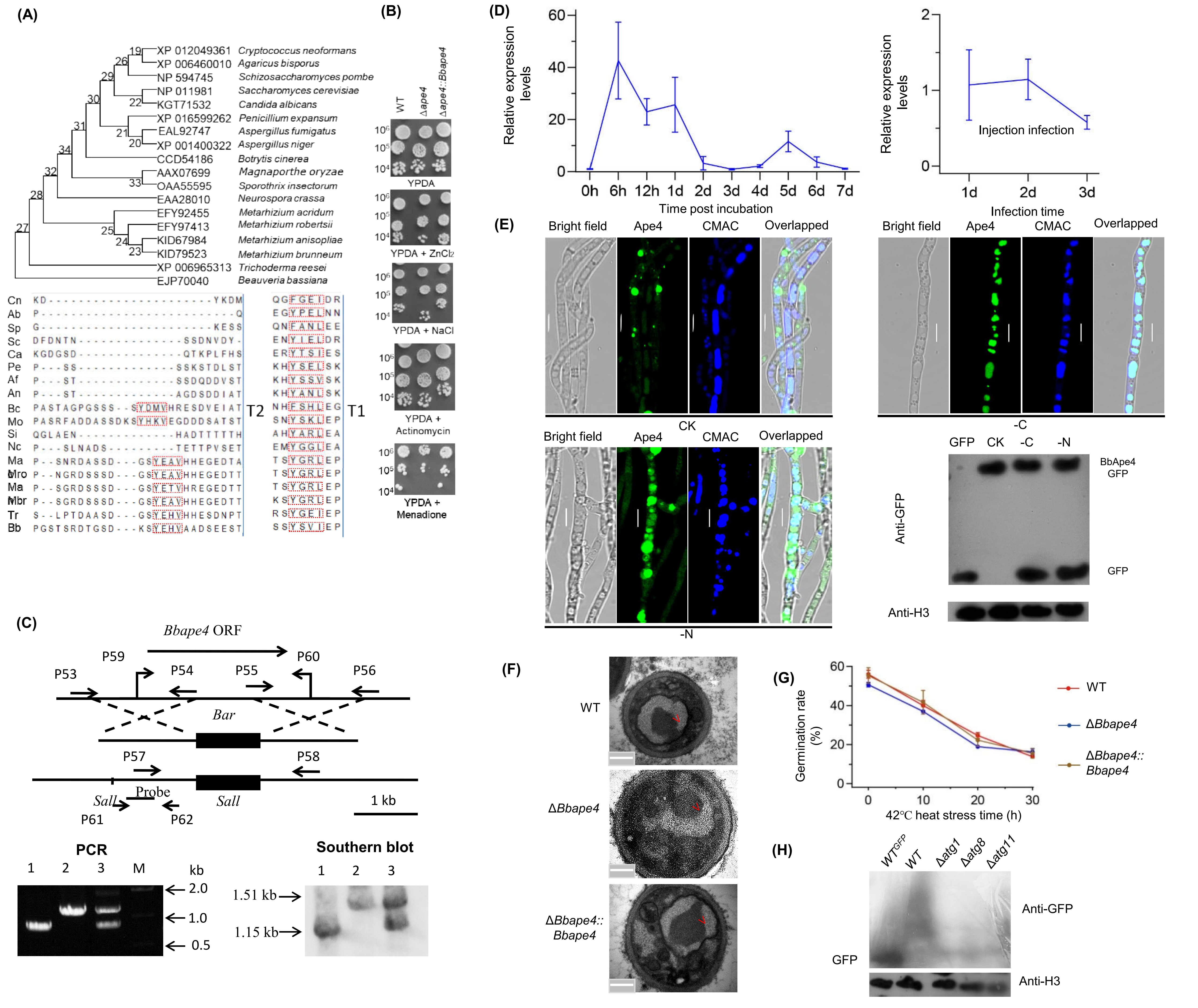

Supplement: FIG S6 [file mbio.03049-22-s0008.pdf]
