# Supplementary material for: The Entomopathogenic Fungus Beauveria bassiana Employs Autophagy as a Persistence and Recovery Mechanism during Conidial Dormancy
Source: mBio. 2023 Feb 21;14(2):e03049-22. doi: 10.1128/mbio.03049-22 (PMC10128008; doi:10.1128/mbio.03049-22)

Fig. S7 Trafficking of Ape4 in *B. bassiana*. (A) Sub-cellular localization of green fluorescent protein in mycelia. The gene GFP was transformed into the wild type. The resulting transformant was inoculated on SDAY/in SDB, and cultured at 25℃. Fungal cells on SDAY plates were sampled at 6 h, 12h, 1 d, 2d, and 3 d post incubation; whereas, the cells in SDB were sampled at 3d post incubation. Green signals were evenly distributed in cytosol in all types of fungal cells. (B) Vacuolar targeting of BbApe4 with different mutated AIM motifs. BbAPE4 with different single mutation (T3–T7) was fused with GFP and transformed into the wild type. Mutation of single motif did not affect the vacuolar targeting of BbApe4. BF: bright field; OL: overlapped. (C) Assay for the interaction of BbApe4 with BbAtg8-β. In yeast two-hybrid (Y2H) test, the yeast strain with BbAtg8-β and BbApe4 did not grow on the SD/-Leu-Trp-Ade-His medium, in contrast to the yeast for positive control (PC) and consist with the negative control (NC). BiFC assay was used to detect the *in vivo* protein interaction. YC-BbATG8-β and YN-BbAPE4 were transformed into the wild-type strain, and the resultant transformant were cultured in SDB for 3 d. The fluorescent signals in mycelia were examined under a laser confocal microscope. No fluorescence was detected in the transformant, which was consistent with the transformant with YC-BbAtg8-β. This result indicated that no interaction is present between BbApe4 and BbAtg8-β. Vacuoles were stained with vacuole-specific dye CMAC. Scale bars: 5 μm.

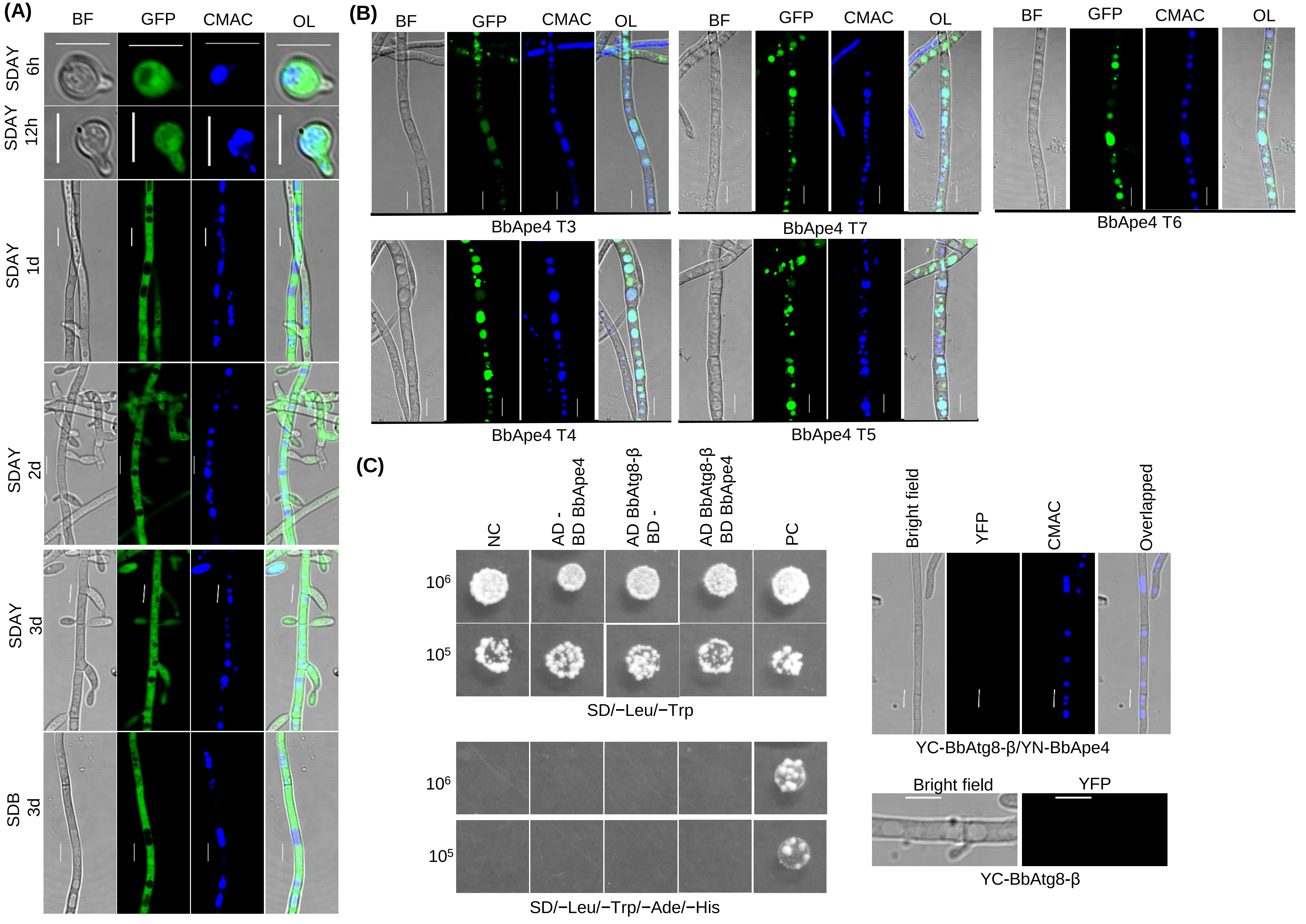

Supplement: FIG S7 [file mbio.03049-22-s0009.pdf]
