# Supplementary material for: The Entomopathogenic Fungus Beauveria bassiana Employs Autophagy as a Persistence and Recovery Mechanism during Conidial Dormancy
Source: mBio. 2023 Feb 21;14(2):e03049-22. doi: 10.1128/mbio.03049-22 (PMC10128008; doi:10.1128/mbio.03049-22)

Fig. S8 Tripeptide at extreme carboxyl-terminus is indispensable for the autophagy role of BbAtg8. The truncated *BbATG8* (*BbATG8^T*) was transformed into the Δ*Bbatg8* mutant strain, and the generated strain was named as Δ*Bbatg8^{A8T}*. (A) Representative images of autophagic bodies. Under transmission electronic microscope, autophagic bodies (yellow arrow) were detected in the vacuole of the wild-type strain, and not in the Δ*Bbatg8* and Δ*Bbatg8^{A8T}* strains. Scale bars: 0.2 μm. (B) Fluorescent view of the autophagic flux. Fused genes of *GFP-BbATG8* and *GFP-BbATG8^T* were integrated into the wild-type strain. Green punctate signals (white arrow) were seen the transformant with *GFP-BbATG8*, and not in the transformant with *GFP-BbATG8^T*. Scale bars: 5 μm; (C) Detecting pexophagy, mitophagy, and Cvt pathway. Peroxisome, mitochondrion, and BbApeI were indicated with GFP. As for pexophagy and mitophagy, fungal strains were cultured in SDB for 2 d, and the resulting mycelia were starved for 3 h. Scale bars: 10 μm. As for Cvt pathway, the strains were cultured on SDAY plates for 12 h. Scale bars: 5 μm. In the wild-type strain, green signals (pink arrow) were translocated into vacuoles. However, no significant green fluorescence was observed in the vacuoles of the Δ*Bbatg8* and Δ*Bbatg8^{A8T}* strains. “BF”: bright field; “OL”: overlapped.

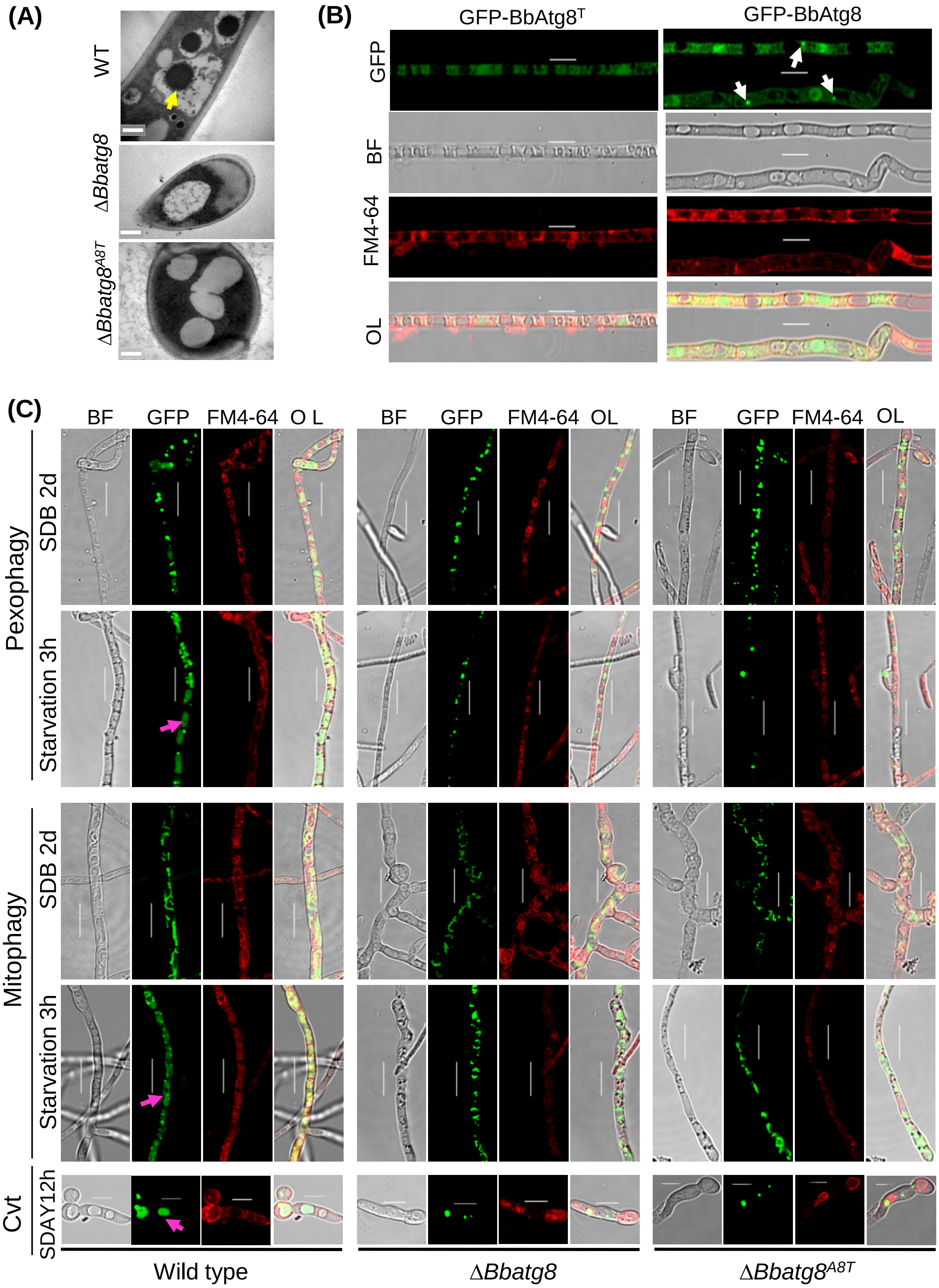

Supplement: FIG S8 [file mbio.03049-22-s0010.pdf]
