# Supplementary material for: The Entomopathogenic Fungus Beauveria bassiana Employs Autophagy as a Persistence and Recovery Mechanism during Conidial Dormancy
Source: mBio. 2023 Feb 21;14(2):e03049-22. doi: 10.1128/mbio.03049-22 (PMC10128008; doi:10.1128/mbio.03049-22)
Supplement: TABLE S2 [file mbio.03049-22-s0002.docx]

**Table S2 Experimental materials used in this study.**

**A: Bacterial and fungal strains.**

| **Strain name** | **Genotype (Plasmid)** | **Reference** |
| --- | --- | --- |
| WT | The wild-type strain | The US Plant, Soil and Nutrition Laboratory |
| Δ*Bbatg1* | *Bbatg1*::*bar* | Ying et al., 2016 |
| Δ*Bbatg1::Bbatg1* | Δ*Bbatg1*(p0380*-sur-BbATG1)* | Ying et al., 2016 |
| Δ*Bbatg8* | *Bbatg8*::*bar* | Ying et al., 2016 |
| Δ*Bbatg8::Bbatg8* | Δ*Bbatg1*(p0380*-sur-BbATG8)* | Ying et al., 2016 |
| ΔB*batg11* | *Bbatg11*::*bar* | Ding et al., 2018 |
| Δ*Bbatg11::Bbatg11* | Δ*Bbatg1*(p0380*-sur-BbATG11)* | Ding et al., 2018 |
| WT^GAtg8^ | WT (p0380-GFP-BbATG8-bar) | This study |
| YH109 | Yeast strain for Y2H assay | Clontech Laboratories, CA, USA |
| pGBKT7-BbAPE4/ pGADT7-Res | *YH109* (pGBKT7-BbAPE4) and (pGADT7- Res) | This study |
| pGADT7-BbATG8 / pGBKT7- Res | *YH109* (pGBKT7- Res) and (pGADT7-BbATG8) | This study |
| pGADT7-BbATG8 / pGBKT7-BbAPE4 | *YH109* (pGADT7-BbATG8) and (pGBKT7-BbAPE4) | This study |
| pGADT7-Res / pGBKT7-Res(Negative Control) | *YH109* (pGADT7- Res) and (pGBKT7- Res) | This study |
| pGADT7-LargeT/pGBKT7-p53(Positive Control) | *YH109* (pGADT7- LargeT) and (pGBKT7- p53) | This study |
| WT^Ape4G^ | WT (p0380-BbAPE4-GFP-sur) | This study |
| WT^3×FlagAtg8(Gfp)^ | WT (p0380- GFP-sur) and (p0380-3×FlagATG8-bar) | This study |
| WT^3×FlagAtg8(Ape4G)^ | WT (p0380-BbAPE4-GFP-sur) and (p0380-3×FlagATG8-bar) | This study |
| BL21 | *E. coli* strainfor Pull-down assay | Novagen |
| BL21^GST-Atg8^ | pGEX 4T-3-BbATG8 | This study |
| BL21^GST^ | pGEX 4T-3 | This study |
| BL2^Ape4HIS^ | pGEX 32-a-BbAPE4 | This study |
| YCBbAtg8  /YN BbApe4 | WT (p0380T-YC-ATG8-B) and (p0380T-APE4-YN-S) | This study |
| YCBbAtg8  /YN - | WT (p0380T-YC-ATG8-B) and (p0380T-YN-S) | This study |
| YC-  /YN BbApe4 | WT (p0380T-YC-B) and (p0380T-APE4-YN-S) | This study |
| YC-  /YN - | WT (p0380T-YC-B) and (p0380T-YN-S) | This study |
| pGADT7-BbAtg8 / pGBKT7-BbApe4 T1 | YH109(pGADT7-BbATG8) and (pGBKT7-BbAPE4 T1) | This study |
| pGADT7-BbAtg8 / pGBKT7-BbApe4 T2 | YH109 (pGADT7-BbATG8) and (pGBKT7-BbAPE4 T2) | This study |
| pGADT7-BbAtg8 / pGBKT7-BbApe4 T3 | YH109(pGADT7-BbATG8) and (pGBKT7-BbAPE4 T3) | This study |
| pGADT7-BbAtg8 / pGBKT7-BbApe4 T4 | YH109(pGADT7-BbATG8) and (pGBKT7-BbAPE4 T4) | This study |
| pGADT7-BbAtg8 / pGBKT7-BbApe4 T5 | YH109(pGADT7-BbATG8) and (pGBKT7-BbAPE4 T5) | This study |
| pGADT7-BbAtg8 / pGBKT7-BbApe4 T6 | YH109 (pGADT7-BbATG8) and (pGBKT7-BbAPE4 T6) | This study |
| pGADT7-BbAtg8 / pGBKT7-BbApe4 T7 | YH109(pGADT7-BbATG8) and (pGBKT7-BbAPE4 T7) | This study |
| WT^Ape4G^ | WT (p0380-BbApe4-GFP-sur) | This study |
| Δ*Bbatg1*^Ape4G^ | Δ*Bbatg1* (p0380-BbApe4-GFP-sur) | This study |
| Δ*Bbatg8*^Ape4G^ | Δ*Bbatg8* (p0380-BbApe4-GFP-sur) | This study |
| Δ*Bbatg11*^Ape4G^ | Δ*Bbatg11* (p0380-BbApe4-GFP-sur) | This study |
| WT^Gfp^ | WT (p0380- GFP-sur) | This study |
| WT^mCherryAtg8(Ape4G)^ | WT (p0380-BbApe4-GFP-sur) and (p0380-mCherry-BbAtg8-bar) | This study |
| *S. cerevisiae*BY4741 | *MATa his3 leu2 met15 ura3* | EUROSCARF, Germany |
| Δ*ape4* | BY4741*ape4*Δ::*kan* | This study |
| Δ*ape4::Bbape4* | Δ*ape4BbApe4*::*kan* | This study |
| Δ*Bbape4* | WT*Bbape4*Δ::*bar* | This study |
| Δ*Bbape4:: Bbape4* | Δ*Bbape4* (p0380*-sur-BbAPE4)* | This study |
| Δ*Bbatg8^A8T^* | Δ*Bbatg8*(p0380*-sur-ATG8^T^*) | This study |
| WT^GAtg8T^ | WT (p0380-GFP-BbATG8^T^-bar) | This study |
| M-WT | WT (pTMTP-mito) | This study |
| M-Δ*Bbatg8* | Δ*Bbatg8* (pTMTP-mito) | This study |
| M-Δ*Bbatg8^A8T^* | Δ*Bbatg8^A8T^* (pTMTP-mito) | This study |
| P-WT | WT (pTMTP-pex) | This study |
| P-Δ*Bbatg8* | *ΔBbatg8* (pTMTP-pex) | This study |
| P-Δ*Bbatg8^A8T^* | Δ*Bbatg8^A8T^* (pTMTP-pex) | This study |
| Ape1-WT | WT (pTMTP- Ape1) | This study |
| Ape1-Δ*Bbatg8* | *ΔBbatg8* (pTMTP- Ape1) | This study |
| Ape1-Δ*Bbatg8^A8T^* | Δ*Bbatg8^A8T^* (pTMTP- Ape1) | This study |
| pGADT7- BbAtg8^T^ / pGBKT7-BbApe4 | YH109 (pGADT7- BbATG8^T^)and (pGBKT7-BbAPE4) | This study |
| pGADT7- BbAtg8^T^ / pGBKT7-Res | YH109 (pGADT7- BbATG8^T^)and (pGBKT7-Res) | This study |
| pGADT7- BbAtg8-β / pGBKT7-BbApe4 | YH109 (pGADT7- BbAtg8-β)and (pGBKT7-BbAPE4) | This study |
| pGADT7- BbAtg8-β/ pGBKT7-Res | YH109 (pGADT7- BbAtg8-β)and (pGBKT7-Res) | This study |
| Δ*Bbatg8^A8T^*^(Ape4G)^ | Δ*Bbatg8^A8T^* (p0380-BbApe4-GFP- ptrA) | This study |
| Δ*Bbape4^T1^* | Δ*Bbape4* (p0380*- BbAPE4^T1^-sur)* | This study |
| Δ*Bbape4^T2^* | Δ*Bbape4* (p0380*- BbAPE4^T2^-sur)* | This study |
| Δ*Bbape4^T3^* | Δ*Bbape4* (p0380*- BbAPE4^T3^-sur)* | This study |
| Δ*Bbape4^T4^* | Δ*Bbape4* (p0380*- BbAPE4^T4^-sur)* | This study |
| Δ*Bbape4^T5^* | Δ*Bbape4* (p0380*- BbAPE4^T5^-sur)* | This study |
| Δ*Bbape4^T6^* | Δ*Bbape4* (p0380*- BbAPE4^T6^-sur)* | This study |
| Δ*Bbape4^T7^* | Δ*Bbape4* (p0380*- BbAPE4^T7^-sur)* | This study |
| WT^Ape4T1G^ | WT (p0380-BbApe4^T1^-GFP-sur) | This study |
| WT^Ape4T2^ | WT (p0380-BbApe4^T2^-GFP-sur) | This study |
| WT^mCherryAtg8(Ape4T1G)^ | WT (p0380-BbApe4^T1^-GFP-sur) and (p0380-mCherry-BbAtg8-bar) | This study |
| WT^mCherryAtg8(Ape4T2)^ | WT (p0380-BbApe4^T2^-GFP-sur) and (p0380-mCherry-BbAtg8-bar) | This study |
| WT^Ape4T3G^ | WT (p0380-BbApe4^T3^-GFP-sur) | This study |
| WT^Ape4T4G^ | WT (p0380-BbApe4^T4^-GFP-sur) | This study |
| WT^Ape4T5G^ | WT (p0380-BbApe4^T5^-GFP-sur) | This study |
| WT ^Ape4T6G^ | WT (p0380-BbApe4^T6^-GFP-sur) | This study |
| WT^Ape4T7G^ | WT (p0380-BbApe4^T7^-GFP-sur) | This study |
| WT^A8^ | WT(p0380-BbAtg8-sur) | This study |
| WT^A8T^ | WT(p0380-BbAtg8^T^-sur) | This study |

**B: Primers for various purposes.**

| **Primers** | **Paired sequences (5′−3′)*** | **Purpose** |
| --- | --- | --- |
| P1/P2 | GCCATGGAGGCCAGTGAATTCATGCGTAGCAAGTTCAAGGATGAGCA/CGCTGCAGGTCGACGGATCCTCAAATGCTGCCAAAGGTGTTCT | Amplifying the *BbATG8* as prey in Y2H |
| P3/P4 | ATGGCCATGGAGGCCGAATTCATGCGTAGCAAGTTCAAGGATGAGCA/CGCTGCAGGTCGACGGATCCTCAAATGCTGCCAAAGGTGTTCT | Amplifying the *BbATG8* as bait in Y2H |
| P5/P6 | ATGGCCATGGAGGCCGAATTCATGGCTCCTCCCCAGGCCGCCCTC/CGCTGCAGGTCGACGGATCCATCAATCAAGATCTTGGGCTCGATGA | Amplifying the *BbAPE4* as bait in Y2H |
| P7P8 | GCCATGGAGGCCAGTGAATTCATGGCTCCTCCCCAGGCCGCCCTC/CGCTGCAGGTCGACGGATCCATCAATCAAGATCTTGGGCTCGATGA | Amplifying the *BbAPE4* as prey in Y2H |
| P9/P10 | ATGGCCATGGAGGCCGAATTCATGGCTCCTCCCCAGGCCGCCC/CGCTGCAGGTCGACGGATCCTCAATCAATCAAGATCTTGGGCTCACCGACGCTGCCCGAGGAATAAA | Mutation of T1 in *BbAPE4* |
| P11/P12 | ATGGCCATGGAGGCCGAATTCATGGCTCCTCCCCAGGCCGCCC/TCGCTGTCGGCTGCACCATGCTCGCCGCTCTTGTCCGAGCCGGTATCGCGGGA | Mutation of T2 in *BbAPE4* |
| P13/P14 | TCCCGCGATACCGGCTCGGACAAGAGCGGCGAGCATGGTGCAGCCGACAGCGA/CGCTGCAGGTCGACGGATCCTCAATCAATCAAGATCTTGGGCT | Mutation of T2 in *BbAPE4* |
| P15/P16 | ATGGCCATGGAGGCCGAATTCATGGCTCCTCCCCAGGCCGCCC/TTTACAGAGGAGATGAGACCCTCACCCGAGCAGCCAGTCATTTCGAGGTTGT | Mutation of T3 in *BbAPE4* |
| P17/P18 | ACAACCTCGAAATGACTGGCTGCTCGGGTGAGGGTCTCATCTCCTCTGTAAA/CGCTGCAGGTCGACGGATCCTCAATCAATCAAGATCTTGGGCT | Mutation of T3 in *BbAPE4* |
| P19/P20 | ATGGCCATGGAGGCCGAATTCATGGCTCCTCCCCAGGCCGCCC/CGGTCGGTCATCTCGGCACCAGGCTCGCCGTCCTCATCACCATCCTCA | Mutation of T4 in *BbAPE4* |
| P21/P22 | TGAGGATGGTGATGAGGACGGCGAGCCTGGTGCCGAGATGACCGACCG/CGCTGCAGGTCGACGGATCCTCAATCAATCAAGATCTTGGGCT | Mutation of T4 in *BbAPE4* |
| P23/P24 | ATGGCCATGGAGGCCGAATTCATGGCTCCTCCCCAGGCCGCCC/CCGCCGCCGTACTTTTCCACGGCACCCTGATCGCCGCCGACCTGCGACTTCT | Mutation of T5 in *BbAPE4* |
| P25/P26 | AGAAGTCGCAGGTCGGCGGCGATCAGGGTGCCGTGGAAAAGTACGGCGGCGG/CGCTGCAGGTCGACGGATCCTCAATCAATCAAGATCTTGGGCT | Mutation of T5 in *BbAPE4* |
| P27/P28 | ATGGCCATGGAGGCCGAATTCATGGCTCCTCCCCAGGCCGCCC/CCCAGGAGTCGCGCTCGTGACCTTGCTGGCCGCCAGCCTTTTCGAAAATC | Mutation of T6 in *BbAPE4* |
| P29/P30 | GATTTTCGAAAAGGCTGGCGGCCAGCAAGGTCACGAGCGCGACTCCTGGG/CGCTGCAGGTCGACGGATCCTCAATCAATCAAGATCTTGGGCT | Mutation of T6 in *BbAPE4* |
| P31/P32 | ATGGCCATGGAGGCCGAATTCATGGCTCCTCCCCAGGCCGCCCTCGATTTTATCGACTTTGTCAATGCCTCGCCGACGCCGGGCCATGCCGGTGCTACCGCCGTC/CGCTGCAGGTCGACGGATCCTCAATCAATCAAGATCTTGGGCT | Mutation of T7 in *BbAPE4* |
| P33/P34 | GCATTCAATCACAAACACCTTCAAAATGGACTACAAGGATCACGACGGCGATTACAAGGATCACGACATCGATTACAAGGATGACGACGATAAGATGCGTAGCAAGTTCAAGGATGAGCA/ AAGCTTGGCTGCAGGTCGACGGATCTCAAATGCTGCCAAAGGTGTTCT | Amplifying 3×Flag BbATG8 fusion fragment |
| P35/P36 | ATACGATGTTCCAGATTACGCTATGCGTAGCAAGTTCAAGGATGAGCA/ AAGCTTGGCTGCAGGTCGACGGATCTCAAATGCTGCCAAAGGTGTTCT | Amplifying the *BbATG8* for BiFC |
| P37/P38 | GCATTCAATCACAAACACCTTCAAAATGGCTCCTCCCCAGGCCGCCCTC/CTCAGAAATCAACTTTTGCTCCATATCAATCAAGATCTTGGGCTCGATGA | Amplifying the *BbAPE4* for BiFC |
| P39/P40 | ATTCAATCACAAACACCTTCAAAATGGCTCCTCCCCAGGCCGCCCTC/CCTGCAGGTCGACGGATCCCCGGGATCAATCAAGATCTTGGGCTCGATGA | Amplifying *BbAPE4* cDNA fragment |
| P41/P42 | ATACGATGTTCCAGATTACGCTATGCGTAGCAAGTTCAAGGATGAGCA/AAGCTTGGCTGCAGGTCGACGGATCTCAAATGCTGCCAAAGGTGTTCT | Amplifying the *BbATG8* cDNA fragment |
| P43/P44 | GCCATGGCTGATATCGGATCCATGGCTCCTCCCCAGGCCGCCCTC/TTGTCGACGGAGCTCGAATTCATCAATCAAGATCTTGGGCTCGATGA | Amplifying *BbAPE4* cDNA fragment for pGEX32-a |
| P45/P46 | GATCTGGTTCCGCGTGGATCCATGCGTAGCAAGTTCAAGGATGAGCA/GGATCCACGCGGAACCAGTCAAATGCTGCCAAAGGTGTTCT | Amplifying the *BbATG8* cDNA fragment for pGEX 4T-3 |
| P47/P48 | TGGGCCCGGCGCGCCGAATTCACGGAGATTCTGGGCTAC/TGGCTGCAGGTCGACGGATCCCGGAAAGTCGGTCTGTAATG | Amplifying 5′-fragment for *BbAPE4* disruption vector |
| P49/P50 | GACCCATGGCTCGAGTCTAGAGAGCAACTTTGACCCGAACC/GTGGCTAGCGTTAACACTAGTCTGGCTTGGTTCAGTTCG | Amplifying 3′-fragment for *BbAPE4* disruption vector |
| P51/P52 | ATCCGTCGACCTGCAGCCAAGCTTTCACGGAGATTCTGGGCTAC/ACACTAGTCAGATCTTCTAGTGTTCCTGGCTTGGTTCAGTTCG | Cloning the *BbAPE4* full ORF for gene complementation |
| P53/P54 | TCTCAAGGTCGCTTCGTC/GCAAGAGGCTCAAAGTCC | PCR detecting *BbAPE4* |
| P55/P56 | ACCATTTCCCGCCACCTGCC/CGGCAACGGCGATGAGATGG | Preparing probes for Southern analysis |
| P57/P58 | ATTCAATCACAAACACCTTCAAAATGGCTCCTCCCCAGGCCGCCC/CCTGCAGGTCGACGGATCCCCGGGTCAATCAATCAAGATCTTGGGCTCACCGACGCTGCCCGAGGAATAAA | Amplifying *BbAPE4^T1^* |
| P59/P60 | ATTCAATCACAAACACCTTCAAAATGGCTCCTCCCCAGGCCGCCCTC/CCTGCAGGTCGACGGATCCCCGGGATCAATCAAGATCTTGGGCTCGATGA | Amplifying *BbAPE4^T2^* |
| P61/P62 | ATTCAATCACAAACACCTTCAAAATGGCTCCTCCCCAGGCCGCCCTC/CCTGCAGGTCGACGGATCCCCGGGATCAATCAAGATCTTGGGCTCGATGA | Amplifying *BbAPE4^T3^* |
| P63/P64 | ATTCAATCACAAACACCTTCAAAATGGCTCCTCCCCAGGCCGCCCTC/CCTGCAGGTCGACGGATCCCCGGGATCAATCAAGATCTTGGGCTCGATGA | Amplifying *BbAPE4^T4^* |
| P65/P66 | ATTCAATCACAAACACCTTCAAAATGGCTCCTCCCCAGGCCGCCCTC/CCTGCAGGTCGACGGATCCCCGGGATCAATCAAGATCTTGGGCTCGATGA | Amplifying *BbAPE4^T5^* |
| P67/P68 | ATTCAATCACAAACACCTTCAAAATGGCTCCTCCCCAGGCCGCCCTC/CCTGCAGGTCGACGGATCCCCGGGATCAATCAAGATCTTGGGCTCGATGA | Amplifying *BbAPE4^T6^* |
| P69/P70 | ATTCAATCACAAACACCTTCAAAATGGCTCCTCCCCAGGCCGCCCTC/CCTGCAGGTCGACGGATCCCCGGGATCAATCAAGATCTTGGGCTCGATGA | Amplifying *BbAPE4^T7^* |
| P71/P72 | TTCAATCACAAACACCTTCAAA*ATGACTACAAGGATCACGACGGCGATTACAAGGATCACGACATCGATTACAAGGATGACGACGATAAG*ATGCGTAGCAAGTTCAAGGA/TAACGTTAAGTGGATCTTA**TTAATGGTGATGGTGATGATGATGGTGATGGTGATGATGATGGTGATGGTGATGATG**TCTCGCCAGAGTAGGTGATATAG | Truncation of *BbATG8* |
| P73/P74 | ATACGATGTTCCAGATTACGCTATGCGTAGCAAGTTCAAGGATGAGCA/AAGCTTGGCTGCAGGTCGACGGATCTCAAATGCTGCCAAAGGTGTTCT | Amplifying the *BbATG8* cDNA fragment |
| P75/P76 | ATACGATGTTCCAGATTACGCTATGCGTAGCAAGTTCAAGGATGAGCA/AAGCTTGGCTGCAGGTCGACGGATCTCTCGCCAGAGTAGGTGATATAG | Amplifying the *BbATG8^T^* cDNA fragment |
| P77/P78 | GTCGACCTGCAGCCAAGCTTGGGCAATTGATTACGGGATC/CGTTAACACTAGTCAGATCTTCTAGAATGGGGTGACGATGAGCCGC | Cloning *ptrA* fragment |
| P79/P80 | ACCATGTTGGGCCCGGCGCGCCTACTGCCGCAAGCAATT/CTGCAGGTCGACGGATCCCCGGGTTTGAAGGTGTTTGTGATTGAA | Cloning TEF promoter promoter |
| P81/P82 | ATGTTGGGCCCGGCGCGCCCCCGGGGATCCACTTAACGTTACTGAA/GGCTGCAGGTCGACGGATCCGAGTGGAGATGTGGAGTGGGCG | Cloning terminator fragment |
| P83/P84 | TTCAATCACAAACACCTTCAAATCATGGTGAGCAAGGGCGAG/TGATTTCAGTAACGTTACAGAGGCGGCTCTTGTACAGCTC | Amplifying the signal peptide of *M3-GFP* fusion fragment |
| P85/P86 | TTCAATCACAAACACCTTCAAAATGGTGAGCAAGGGCGAGGA/TGATTTCAGTAACGTTAAGTGGATCCTCAGAGGCGGCTCTTGTACAGCTCGTCCATG | Constructing a GFP-SRL fragment |
| P87/P88 | GCCATGGAGGCCAGTGAATTCATGCGTAGCAAGTTCAAGGATGAGCA/ CGCTGCAGGTCGACGGATCCTCTCGCCAGAGTAGGTGATATAG | Amplifying the *BbATG8^T^* as prey in Y2H |
| P89/P90 | ATTCAATCACAAACACCTTCAAAATGGCTCCTCCCCAGGCCGCCCTC/AGTAACGTTAAGTGGATCGTCGACTTACTTGTACAGCTCGTCCATGCCG | Amplifying the *BbAPE4-GFP* fusion fragment |
| P81/P82 | GCCATGGAGGCCAGTGAATTCATGCGTAGCAAGTTCAAGGATGAGCA/CGCTGCAGGTCGACGGATCCTCTCGCCAGAGTAGGTGATATAG | Amplifying the *BbATG8-β*as prey in Y2H |
| P91/P92 | CTGTATGAATGAAAGAAAAAATTCATGTTATGTGAGCAAGTAGGTAAAAAGAAAGAATAAAAGGTTCAGCTTTTCTAATGTTTCTACTCCTTTTTTACTC/TATATATTTACTATTTGTAAAATACAAAAGAGGGCAATAAATATACCGGATTATGAATCATCTAACAATTGCAGTCATTTAGACAACAATTTCAGATTCTATG | Amplifying fragment for disruption of *APE4* in yeast BY4741 |
| P93/P94 | CCTGTATGAATGAAAGAAAAAATTCATGTTATGTGAGCAAGTAGGTAAAAAGAAAGAATAAAAGGTTCAGCTTTTCTATGGCTCCTCCCCAGGCCGCCCT/ATTTCAGTAACGTTAAGTGGATCTCAATCAATCAAGATCTTGGGC | Amplifying APE4 promoter for complementation of Δ*ape4* |
| P95/P96 | GCCCAAGATCTTGATTGATTGAGATCCACTTAACGTTACTGAAAT/AGAGTAAAAAAGGAGTAGAAACATTGAGTGGAGATGTGGAGTGGGCGC | Amplifying *BbAPE4* for complementation of Δ*ape4* |
| P97/P98 | GCGCCCACTCCACATCTCCACTCAATGTTTCTACTCCTTTTTTACTC/ TATATATTTACTATTTGTAAAATACAAAAGAGGGCAATAAATATACCGGATTATGAATCATCTAACAATTGCAGTCATTCAAATATGTATCCGCTCATGAA | Amplifying Kan cassette for complementation of Δ*ape4* |
| P99/P100 | TCAAGGAGAATCTATACG/ATGAGGTTGATATGTGTA | qRT-PCR for *BbATG1* |
| P101/P102 | ATAAGAAGAAGTATCTCGTA/GACAAAGATGAAGATAGC | qRT-PCR for *BbATG8* |
| P103/P104 | GGATGGATAGTAGCAATG/GATTCTGTGGTAGATTGTAT | qRT-PCR for *BbATG11* |
| P105/P106 | CAACGACAACACCATCCGCAT/CTCCTCTGTAAATGCCAAG | qRT-PCR for *BbAPE4* |
| P107/P108 | TGGTTTCTAGGACCGCCGTAA/CCTTGGCAAATGCTTTCGC | Reference in qRT-PCR |

*: The underlined sequences are required for integrating the DNA fragments into vectors by homologous recombination. The sequences with double underline are the introduced mutation. The italic region in P71 is the coding sequences for Flag tag, and the bold region in P72 is the coding sequence for His tag.
