# Supplementary material for: The Entomopathogenic Fungus Beauveria bassiana Employs Autophagy as a Persistence and Recovery Mechanism during Conidial Dormancy
Source: mBio. 2023 Feb 21;14(2):e03049-22. doi: 10.1128/mbio.03049-22 (PMC10128008; doi:10.1128/mbio.03049-22)

Fig. S3 Phenotypic evaluation. Conidia of the indicated strain were stored at 25°C and sampled at an interval of 7 days up to four weeks. (A, D, G) Conidial germination. The sampled conidia were inoculated on water agarose (WA), nutrient-rich (SPA), and menadione-stress (SPA + menadione) plates. After 24 h of incubation at 25°C, conidial germination was determined and indicated as germination percentage (%). Two types of bioassay methods (i.e., intrahemoceol injection and cuticle penetration) were used to determine conidial virulence. (B, E, H) Survival trends (%) of the infected insect host over bioassay time. The statistical difference between the paired curves was calculated by the log-rank test and was considered to be significant at *P*<0.05. (C, F, I) Median lethal time (LT$_{50}$) was calculated by the Kaplan-Meier method for three independent bioassays of conidia at the indicated age point. "ND": no LT$_{50}$ value was determined due to their mortalities less than 50%. (A, B, C) Assays for the complementation mutant strains. Δ*Bbatg1*, Δ*Bbatg8*, and Δ*Bbatg11* mutant strains were complemented by ectopic insertion of their entire genes. (D, E, F) Functionality of mutated BbApe4 was determined in Δ*Bbape4* mutant strain, using the wild-type strain as positive control. T3–T7: BbApe4 with single mutation at different motifs (T3–T7). (G, H, I) Phenotypic assays for the strain over-expressing the gene *BbATG8* or *BbATG8*^{T}. *BbATG8* and truncated *BbATG8* (*BbATG8*^{T}) was transformed into the wild-type strain (WT), and the resultant strains were named as WT^{A8} and WT^{A8T}, respectively.

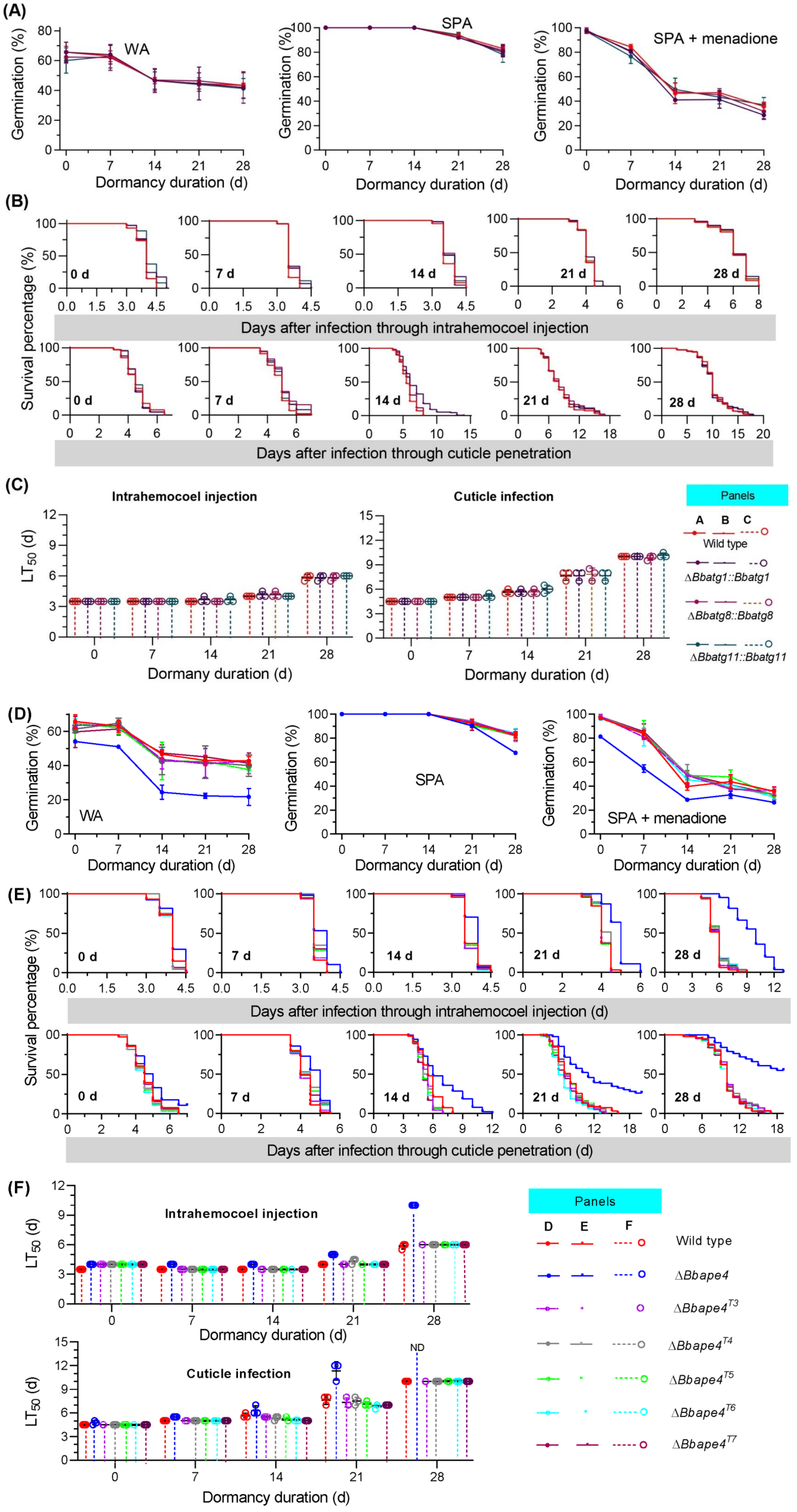

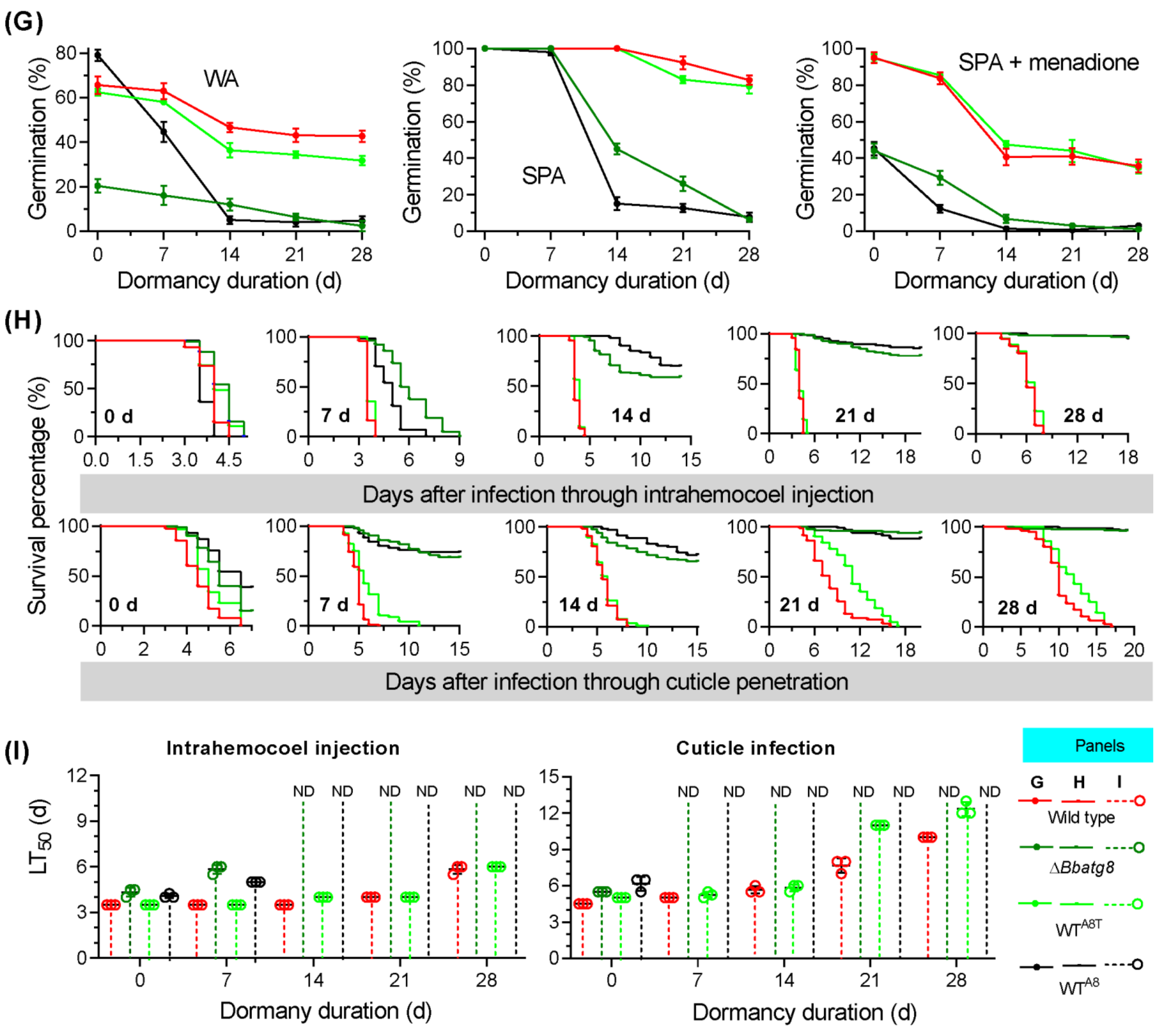

Supplement: FIG S3 [file mbio.03049-22-s0005.pdf]
